# Associations of non-traditional cardiovascular risk factors and body mass index with metabolic syndrome in the Chinese elderly population

**DOI:** 10.1186/s13098-023-01047-4

**Published:** 2023-06-16

**Authors:** Aijun You, Yaxin Li, Chaonan Shen, Huimin Fan, Jia He, Zhongmin Liu, Qian Xue, Yuzhen Zhang, Liang Zheng

**Affiliations:** 1 Department of Epidemiology and Public Health, Tongji University School of Medicine, Shanghai, 200092 China; 2 Research Center for Translational Medicine, Shanghai East Hospital, Tongji University School of Medicine, Shanghai, 200120 China; 3 Institute of Integrated Traditional Chinese and Western Medicine for Cardiovascular Chronic Diseases, Tongji University School of Medicine, Shanghai, 200120 China; 4 Department of Health Statistics, Faculty of Health Service, Naval Medical University, Shanghai, 200433 China; 5 Changhai Clinical Research Unit, Shanghai Changhai Hospital, Naval Medical University, Shanghai, 200433 China

**Keywords:** Cardiovascular disease, Non-traditional cardiovascular risk factors, Metabolic syndrome, Body mass index, The elderly population

## Abstract

**Background:**

Metabolic syndrome (MetS), a clustering of traditional cardiovascular risk factors (CVRF), is currently one of the major global public health burdens. However, associations between MetS and non-traditional CVRF represented by uric acid (UA), homocysteine (HCY) and hypersensitive C-reactive protein (HsCRP) have not been well explored in the elderly population, especially when considering body mass index (BMI).

**Methods:**

Participants from the Shanghai Elderly Cardiovascular Health (SHECH) study cohort in 2017 were analyzed. MetS was defined using the modified American Heart Association/National Heart, Lung, and Blood Institute Scientific Statement. Logistic regression models were used to assess associations of non-traditional CVRF, BMI with MetS.

**Results:**

Of the 4360 participants analyzed, 2378 (54.5%) had MetS, the mean (SD) UA was 331 (86) µmol/L, and the median (IQR) HCY and HsCRP were 15 (13–18) µmol/L and 1.0 (0.5–2.1) mg/L, respectively. Participants with higher non-traditional CVRF tended to have a higher significant risk of MetS (*P* < 0.001), which did not changed substantially in most population subgroups (*P*-interaction > 0.05). BMI mediated 43.89% (95%CI: 30.38–57.40%), 37.34% (95% CI: 13.86–60.83%) and 30.99% (95%CI: 13.16–48.83%) of associations of hyperuricemia (HUA), hyperhomocysteinemia (HHCY) and high HsCRP (HHsCRP) with MetS, respectively. Abnormal non-traditional CVRF combined with overweight/obesity greatly increased MetS risk (adjusted OR(95%CI): HUA + Overweight: 5.860(4.059-8.461); 6.148(3.707–10.194); HHCY + Overweight: 3.989(3.107-5.121); HHCY + Obese: 5.746(4.064–8.123); HHsCRP + Overweight: 4.026(2.906-5.580); HHsCRP + Obese: 7.717(4.508–13.210)).

**Conclusions:**

In the Chinese elderly population, HUA, HHCY, and HHsCRP were all significantly and independently associated with MetS, supporting the potential of focusing on non-traditional CVRF interventions for preventing and controlling MetS. BMI played moderate mediating roles in associations between non-traditional CVRF and MetS, and abnormal non-traditional CVRF combined with overweight/obesity had significant synergistic effects on MetS risk, highlighting the importance of better weight management in the elderly population.

**Supplementary Information:**

The online version contains supplementary material available at 10.1186/s13098-023-01047-4.

## Introduction

Metabolic syndrome (MetS) is currently one of the major global public health burdens [1]. MetS is a clustering of metabolic and cardiovascular risk factors (CVRF), generally including abdominal obesity, hypertension, hyperglycemia, and atherogenic dyslipidemia [2], that could increase the risk of cardiovascular disease (CVD) and adverse outcomes not only through internal single components but also through multiple interacting states [3]. Epidemiological studies have found that compared to non-MetS individuals, those with MetS had a 3-fold increased risk of diabetes mellitus [4], a 2-fold increased risk of cardiovascular morbidity and mortality [5], a 1.5-fold increased risk of all-cause mortality [6], and a significant association with cancers [7]. Furthermore, the incidence and prevalence of this disease is increasing globally with socioeconomic development, lifestyle changes, and especially population aging, affecting approximately one-fifth of the adult population in the Asia-Pacific region [8]. In China, the age-specific prevalence of MetS is approximately 14%, and is up to 30–40% in those aged 60 years and older [9, 10]. Therefore, active prevention and control of MetS is crucial to improve quality of life for the elderly population.

Alternatively, to improve the science of cardiovascular health management, numerous studies have relied on MetS components to develop various cardiovascular risk assessment tools, such as the Framingham Risk Score, the Pooled Cohort Equation, and the China-PAR model, which have suggested that the 10-year cardiovascular risk increases substantially with increasing numbers of MetS components, resulting in individuals being more likely to be classified as high risk for clinical intervention or pharmacological treatment [11–15]. However, relying solely on the results of MetS component assessments, particularly in the elderly population, might not be appropriate [16, 17]. Therefore, an increasing number of studies have focused on the complementary role of non-traditional CVRF in the assessment of CVD risk by MetS components [18–21].

Hyperuricemia (HUA) [22, 23], hyperhomocysteinemia (HHCY) [24, 25] and high hypersensitive C-reactive protein (HHsCRP) (chronic inflammation) [26]were commonly included in the defining extents of MetS, probably due to the association of these disorders with insulin resistance, the major underlying mechanism of MetS. However, the association of these disorders with MetS in the advanced age group has not been well established, as uric acid (UA) [27], homocysteine (HCY)[28] and HsCRP [29] all change with age. Furthermore, the above disorders are representative non-traditional risk factors for CVD, and significant independent associations with CVD are frequently observed and consequently are commonly included in traditional cardiovascular risk assessment tools, but the results of these studies suggested that the incremental effect of non-traditional CVRF on traditional risk assessment lacked sufficient validity [18–21]. This might be because, beyond comprising independent risk factors for CVD, the interaction with MetS components was controversial [30, 31].

Abdominal obesity is a core component of MetS, so one of the easiest ways to actively prevent and manage MetS is to lose weight [32]. Abdominal obesity is typically used as a measure of the individual’s visceral fat accumulation, and the visual response is more focused on the individual’s waist and abdomen [33]. Body mass index (BMI) is more typically used to reflect an individual’s overall obesity condition, even in the absence of abdominal obesity, and BMI has been demonstrated to be associated with CVRF and CVD [34]. However, an increasing number of studies have found a significant reduction in CVD risk when individuals were metabolically healthy and obese [35, 36]; additionally, overweight/obesity as assessed by BMI has been found to be contradictory to mortality in the advanced age group, i.e., the “obesity paradox“ [37, 38]. Thus, the association direction between BMI in traditional and non-traditional CVRF might be altered in the elderly population.

Accordingly, using database from the Shanghai Elderly Cardiovascular Health (SHECH) study, this study aimed to investigate (1) the independent association of UA, HCY and HsCRP (non-traditional CVRF) with MetS; (2) to assess the mediating role of BMI in the association of non-traditional CVRF with MetS; and (3) to explore the synergistic effect of the combined abnormal status of non-traditional CVRF and BMI on MetS.

## Methods

### Study population

Participants were recruited from the SHECH study, a population-based longitudinal study assessing risk factors for the onset and progression of CVD in noninstitutionalized older adults since 2013. Its design details have been described previously [39]. Briefly, 4753 community participants aged 60 to 104 years were recruited during 2017 and detailed health screenings and questionnaires were administered to these participants. In this study, 393 participants were excluded due to missing MetS, thus 4360 participants were finally included (Supplementary Fig. 1).

### Data collection

Baseline characteristics of participants were collected by trained investigators using a standardized questionnaire that included primarily sociodemographic, lifestyle, medication, and disease history. Anthropometric measurements including height, body weight, and waist circumference (WC) were performed according to the recommendations of International Standards for Anthropometric Assessment [40]. Medication and disease histories were obtained through participant self-report and confirmed by family physicians making best efforts to review outpatient medical records to minimize recall bias. Blood pressure (BP) and blood sample acquisition and measurement have been described in detail in the published literature [39]. Biochemical parameters included creatinine, UA, HCY, HsCRP, thyroid stimulating hormone (TSH), aspartate aminotransferase (AST), alanine aminotransferase (ALT), glucose, total cholesterol (TC), triglycerides (TG), LDL-C and high-density lipoprotein cholesterol (HDL-C), and all these were measured using a biochemical autoanalyzer (Cobas 8000, Roche Diagnostics, Mannheim, Germany) under standardized operating procedures.

Celibacy was considered active if an individual was currently unmarried, divorced, or widowed. Current smoking was defined as smoking more than 100 cigarettes in a lifetime and still smoking. Current drinking was defined as drinking 1 or more alcoholic beverages each week during the previous year. Physical activity was considered active if at least 4 days of exercise or recreational activities were performed per week and more than 30 min per day. Coffee/tea addiction was defined as consuming at least one cup of coffee or tea per day for the past month. BMI was calculated as weight in kilograms divided by height in meters squared and was classified as underweight (< 18.5 kg/m^2^), normal weight (18.5-23.9 kg/m^2^), overweight (24.0-27.9 kg/m^2^) and obese (≥ 28.0 kg/m^2^) according to Chinese standards [41].

HUA was defined as UA greater than 420 µmol/L for both genders, given that all participants were of advanced age [42]. HHCY was defined as HCY greater than 15 µmol/L [43]. HHsCRP was defined as HsCRP greater than 3.0 mg/L [44]. Arteriosclerotic CVD (ASCVD) was defined as a history of myocardial infarction, stable or unstable angina, coronary or other arterial revascularization, stroke, transient ischemic attack, or atherosclerotic peripheral artery disease [11]. Thyroid dysfunction was defined as TSH outside the reference range (0.27–4.20 µIU/mL) or a history of thyroid disease. Liver dysfunction was defined as AST greater than 40 U/L, ALT greater than 50 U/L or a history of liver disease. Kidney dysfunction was defined as estimated glomerular filtration rate (GFR) less than 60 mL/min/1.73m^2^ or a history of kidney disease [45].

### Outcome ascertainment

In this study, MetS was defined using the modified American Heart Association/National Heart, Lung, and Blood Institute Scientific Statement [2]. Participants were diagnosed with MetS if three or more of the following disorders were present: (1) abdominal obesity: WC ≥ 90 cm in males and ≥ 80 cm in females; (2) hyperglycemia: fasting plasma glucose (FPG) ≥ 5.6 mmol/L (100 mg/dL) or medication for elevated glucose; (3) hypertension: BP ≥ 130/85 mmHg or antihypertensive medications in patients with a history of hypertension; (4) fasting TG ≥ 150 mg/dL (1.7 mmol/L) or medication for elevated TG; and (5) fasting HDL-C < 40 mg/dL (1.03 mmol/L) in males and < 50 mg/dL (1.30 mmol/L) in females, or medication for reduced HDL-C.

### Statistical analysis

Normality tests were initially performed for continuous variables, and natural logarithm (Ln) transformations were applied to variables with severely skewed distributions.

Continuous variables were reported as mean (standard deviation, SD) or median (interquartile range, IQR) as appropriate, and differences were compared using independent samples t-test or Mann-Whitney U-test. Categorical variables were reported as frequency (%), and differences were compared using Pearson’s χ^2^ test or Fisher’s exact test. Spearman’s rank correlation coefficients (*ρ*) were calculated to initially assess strength of associations of non-traditional CVRF and BMI with MetS.

Logistic regression models were used to assess associations of non-traditional CVRF with MetS and to calculate odds ratios (OR) and 95% confidence intervals (CI). In the model, non-traditional CVRF was fully explored at the ordinal (lowest tertile as reference), binary (none as reference), and continuous (Per SD increment), scale, and adjusted for confounders potentially affecting associations according to previous studies [46, 47]. The 10th, 50th, and 90th percentiles of UA, Ln HCY, and Ln HsCRP were used as knots in restricted cubic splines to simulate possible nonlinear associations of non-traditional CVRF with MetS [48].

To explain mediating effects of BMI in associations of non-traditional CVRF with MetS, causal mediation analyses were performed in a general counterfactual framework that provides clear definitions of causal mediation and associated effects [49]. Under this framework, total effect could be decomposed into: natural direct effect (NDE) and natural indirect effect (NIE), both measured as OR(95%CI) [50]. NDE represented effects of non-traditional CVRF on MetS independent of BMI; NIE represented effects of non-traditional CVRF on MetS explained by BMI. Mediating effects were measured by percentage mediated (95%CI), as the percentage of total effect mediated by the mediator.

To assess joint associations of non-traditional CVRF and BMI with MetS, participants were further divided into eight groups according to non-traditional CVRF (normal/abnormal) and ordinal BMI (underweight/normal weight/overweight/obese) and OR (95% CI) for MetS was estimated in different groups compared to those with normal status of non-traditional CVRF and BMI. Considering ordinal BMI, interactions of abnormal status of non-traditional CVRF and BMI were more interested. For multiplicative interactions, the product term of both was additionally included in the model, and OR (95% CI) of the product term was used as measure of interaction at the multiplicative scale. For additive interactions, relative excess risk due to interaction (RERI) (95% CI) was used as measure of interactions at the additive scale, calculated using coefficients and corresponding standard errors of both exposed and product terms, as well as covariance matrix [51].

The primary analyses were also performed in demographic subgroups, including age group (< 75 yrs/≥ 75 yrs, defined as advanced age by the World Health Organization [52]), gender (male/female), occupation (office staff/operator/farmer/other), education (≤ 6 yrs/6-12 yrs/> 12 yrs), and monthly income (≤ 2500 ¥/> 2500 ¥), to examine the robustness and possible variations of findings.

All statistical analyses were performed using SAS, version 9.4 (SAS Institute, Inc., Cary, NC, USA). Two-sided *P*-values < 0.05 were considered statistically significant.

## Results

### Baseline characteristics

The mean age (SD) of the 4360 participants was 72 (7) years, 1939 (44.5%) were male, 1013 (23.2%) were farmers, and 2349 (53.9%) had more than 2500 ¥ monthly income. The mean (SD) UA was 331 (86) µmol/L, and the median (IQR) HCY and HsCRP were 15 (13–18) µmol/L and 1.0 (0.5–2.1) mg/L, respectively. 2378 (54.5%) participants were found to have MetS, and the prevalence of MetS was higher among participants who were current smokers, current alcohol drinkers, and had ASCVD, liver, kidney and thyroid dysfunction (P <  0.05) (Table 1).

**Table 1:** Baseline characteristics of participants

Characteristic	Total (n = 4360)	Non-MetS (n = 1982)	MetS (n = 2378)	*P*-value
**Demographics**				
Age (yrs)	72 (7)	72(7)	72(7)	0.521
Age group (yrs)				0.156
< 75	3099(71.1)	1390(70.1)	1709(71.9)	
≥ 75	1230(28.2)	581(29.3)	649(27.3)	
Male	1939(44.5)	1045(52.7)	894(37.6)	< 0.001
Celibacy	718(16.5)	311(15.7)	407(17.1)	0.153
Occupation				0.023
Office staff	638(14.6)	310(15.6)	328(13.8)	
Operator	1279(29.3)	618(31.2)	661(27.8)	
Farmer	1013(23.2)	445(22.5)	568(23.9)	
Other	1132(26.0)	490(24.7)	642(27.0)	
Education (yrs)				0.042
≤ 6	1311(30.1)	566(28.6)	745(31.3)	
6–12	2302(52.8)	1071(54.0)	1231(51.8)	
> 12	480(11.0)	236(11.9)	244(10.3)	
Income (¥/month)				0.002
≤ 2500	1572(36.1)	667(33.7)	905(38.1)	
> 2500	2349(53.9)	1115(56.3)	1234(51.9)	
**Lifestyle**				
Current smoking	523(12.0)	308(15.5)	215(9.0)	< 0.001
Current drinking	370(8.5)	206(10.4)	164(6.9)	< 0.001
Physical activity	3425(78.6)	1533(77.3)	1892(79.6)	0.051
Multivitamin intake	61(1.4)	25(1.3)	36(1.5)	0.347
Coffee/tea addiction	322(7.4)	153(7.7)	169(7.1)	0.512
**Medication History**				
Hypolipidemic drugs	484(11.1)	36(1.8)	448(18.8)	< 0.001
Hypotensive drugs	2306(52.9)	737(37.2)	1569(66.0)	< 0.001
Hypoglycemic drugs	637(14.6)	107(5.4)	530(22.3)	< 0.001
**Comorbidity**				
Abdominal obesity	2693(61.8)	795(40.1)	1898(79.8)	< 0.001
Hyperglycemia	1892(43.4)	330(16.6)	1562(65.7)	< 0.001
Hypertension	3690(84.6)	1438(72.6)	2252(94.7)	< 0.001
High TG	1880(43.1)	212(10.7)	1668(70.1)	< 0.001
Low HDL-C	1482(34.0)	94(4.7)	1388(58.4)	< 0.001
HHCY	2088(47.9)	939(47.4)	1149(48.3)	0.420
HHsCRP	699(16.0)	261(13.2)	438(18.4)	< 0.001
HUA	636(14.6)	232(11.7)	404(17.0)	< 0.001
ASCVD	561(12.9)	168(8.5)	393(16.5)	< 0.001
Liver dysfunction	215(4.9)	69(3.5)	146(6.1)	< 0.001
Kidney dysfunction	213(4.9)	79(4.0)	134(5.6)	0.012
Thyroid dysfunction	1030(23.6)	435(21.9)	595(25.0)	0.008
**Physical Examination**				
BMI (kg/m^2^)	24.7 (3.5)	23.5 (3.4)	25.7 (3.3)	< 0.001
WC (cm)	88(12)	84(12)	91(12)	< 0.001
SBP (mmHg)	142 (21)	138 (21)	145 (20)	< 0.001
DBP (mmHg)	80 (11)	79(11)	81 (11)	< 0.001
TC (mg/dL)	192 (49)	193 (35)	192 (58)	0.641
TG (mg/dL)	126(92–174)	101(79–127)	162(117–213)	< 0.001
HDL-C (mg/dL)	53(44–64)	60(52–71)	47(40–56)	< 0.001
LDL-C (mg/dL)	127(37)	126 (32)	127 (41)	0.221
FPG (mmol/L)	5.4(4.9–6.3)	5.1(4.8–5.4)	5.9(5.2–7.0)	< 0.001
HCY (µmol/L)	15(13–18)	15(13–18)	15(13–18)	0.814
ALT (U/L)	16(12–21)	14(11–19)	17(13–24)	< 0.001
AST (U/L)	20(18–24)	21(18–24)	20(17–24)	0.090
GFR (mL/min/1.73 m^2^)	102(23)	102(22)	102(23)	0.700
HsCRP (mg/L)	1.0(0.5–2.1)	0.8(0.4–1.7)	1.1(0.6–2.3)	< 0.001
UA (µmol/L)	331(86)	321(83)	340(87)	< 0.001
TSH (µIU/mL)	2.73(1.91–3.89)	2.62(1.83–3.76)	2.81(1.99–3.99)	< 0.001

Note: Data are mean (standard deviation), median (interquartile range), or frequency (%)

Abbreviations: MetS, metabolic syndrome; HHCY, hyperhomocysteinemia; HHsCRP, high hypersensitive C-reactive protein; HUA, hyperuricemia; ASCVD, arteriosclerotic cardiovascular disease; BMI, body mass index; WC, waist circumference; SBP, systolic blood pressure; DBP, diastolic blood pressure; TC, total cholesterol; TG, triglyceride; HDL-C, high-density lipid cholesterol; LDL-C, low-density lipid cholesterol; FPG; fasting plasma glucose; HCY, homocysteine; ALT, alanine aminotransferase; AST, aspartate aminotransferase; GFR, glomerular filtration rate; UA, uric acid; TSH, thyroid stimulating hormone

### Associations of non-traditional CVRF with MetS

UA was significantly correlated with traditional CVRF (*P* < 0.001), except for FPG (*ρ* = 0.005, *P* = 0.756), and positively correlated with number of MetS components (*ρ* = 0.141, *P* < 0.001). No significant correlations were found for HCY with FPG, DBP, TG and number of MetS components (*P* > 0.05). HsCRP was significantly positively correlated with traditional CVRF (*P* < 0.001), except for HDL-C (*ρ* = -0.217, *P* < 0.001). BMI presented moderate correlation with WC (*ρ* = 0.587, *P* < 0.001), standardized score of MetS (*ρ* = 0.465, *P* < 0.001) and number of MetS components (*ρ* = 0.407, *P* < 0.001). Furthermore, there were significant positive correlations of BMI with UA (*ρ* = 0.178, *P* < 0.001), HCY (*ρ* = 0.047, *P* = 0.001) and HsCRP (*ρ* = 0.200, *P* < 0.001) (Supplementary Table 1). Although correlations between traditional and non-traditional CVRF were found, most were relatively weak and therefore required further analyses.

In the adjusted model, abnormal non-traditional CVRF was significantly associated with MetS. Specifically, MetS risk was significantly increased by 88.4% (95%CI: 1.513–2.345) in HUA than non-HUA; by 32.1% (95%CI: 1.127–1.549) in HHCY than non-HHCY; and by 48.2% (95%CI: 1.212–1.812) for HHsCRP than non-HHsCRP. The adjusted logistic regression model at the continuous scale showed no substantial changes, except for Ln HCY (OR(95%CI): 1.074(0.990-1.165)) (Table 2).

**Table 2:** Association of non-traditional CVRF with MetS

	Unadjusted model		Adjusted model	
	OR(95%CI)	*P*-value	OR(95%CI)	*P*-value
HUA (vs. Non-HUA)	1.555(1.307–1.850)	< 0.001	1.884(1.513–2.345)	< 0.001
Per SD in UA	1.254(1.179–1.333)	< 0.001	1.433(1.321–1.554)	< 0.001
UA (µmol/L, median)				
Tertile 1 (222)	1.000 [ref.]		1.000 [ref.]	
Tertile 2 (306)	1.363(1.177–1.578)	< 0.001	1.588(1.325–1.902)	< 0.001
Tertile 3 (382)	1.751(1.511–2.029)	< 0.001	2.332(1.926–2.824)	< 0.001
Trend		< 0.001		< 0.001
HHCY (vs. Non-HHCY)	1.050(0.932–1.184)	0.420	1.321(1.127–1.549)	< 0.001
Per SD in Ln HCY	0.987(0.930–1.048)	0.668	1.074(0.990–1.165)	0.086
HCY (µmol/L, median)				
Tertile 1 (12)	1.000 [ref.]		1.000 [ref.]	
Tertile 2 (15)	0.984(0.850–1.140)	0.833	1.184(0.988–1.420)	0.067
Tertile 3 (20)	1.009(0.871–1.168)	0.908	1.302(1.068–1.586)	0.009
Trend		0.870		0.012
HHsCRP (vs. Non-HHsCRP)	1.448(1.225–1.712)	< 0.001	1.482(1.212–1.812)	< 0.001
Per SD in Ln HsCRP	1.339(1.256–1.426)	< 0.001	1.321(1.224–1.426)	< 0.001
HsCRP (mg/L, median)				
Tertile 1 (0.4)	1.000 [ref.]		1.000 [ref.]	
Tertile 2 (1.0)	1.778(1.530–2.067)	< 0.001	1.551(1.294–1.860)	< 0.001
Tertile 3 (3.0)	2.117(1.819–2.465)	< 0.001	1.993(1.659–2.394)	< 0.001
Trend		< 0.001		< 0.001

Note: Adjusted for age group, gender, occupation, education, monthly income, current smoking, current drinking, physical activity, ASCVD, liver, kidney, and thyroid dysfunction

Abbreviations: CVRF, cardiovascular risk factors; MetS, metabolic syndrome; UA, uric acid; HUA, hyperuricemia; Ln HCY, natural logarithm of homocysteine; HHCY, hyperhomocysteinemia; Ln HsCRP, natural logarithm of hypersensitive C-reactive protein; HHsCRP, high hypersensitive C-reactive protein; ASCVD, arteriosclerotic cardiovascular disease; SD, standard deviation; OR, odds ratio; CI, confidence interval

**Fig. 1:**
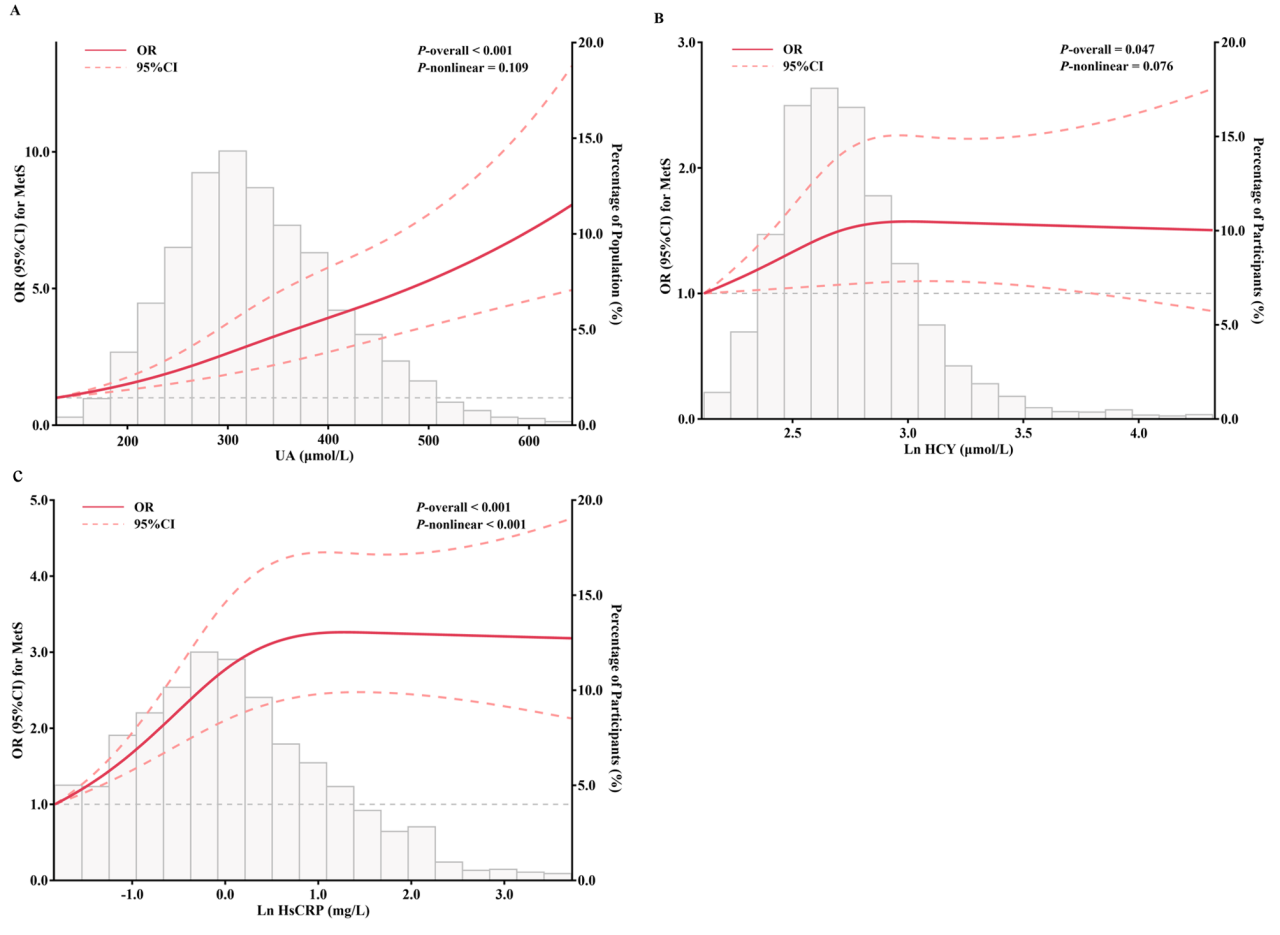
Nonlinear associations of non-traditional CVRF with MetS. **A: UA; B: Ln HCY; C: Ln HsCRP.** Knots were at the 10th, 50th, and 90th percentiles of the UA, Ln HCY and Ln HsCRP distribution. Default reference points were the medians of UA, Ln HCY and Ln HsCRP, respectively. Adjusted for age group, gender, occupation, education, monthly income, current smoking, current drinking, physical activity, ASCVD, liver, kidney, and thyroid dysfunction. Abbreviations: CVRF, cardiovascular risk factors; MetS, metabolic syndrome; UA, uric acid; Ln HCY, natural logarithm of homocysteine; Ln HsCRP, natural logarithm of hypersensitive C-reactive protein; ASCVD, arteriosclerotic cardiovascular disease; OR, odds ratio; CI, confidence interval

The splines between non-traditional CVRF and MetS showed that UA (*P*-nonlinear = 0.109) and Ln HCY (*P*-nonlinear = 0.076) were not significantly nonlinearly associated with MetS, except for Ln HsCRP (*P*-Pnonlinear<  0.001). MetS risk increased with increasing UA, but MetS risk did not change substantially with increasing Ln HCY and Ln HsCRP to approximately 3.0 μmol/L and 1.0 mg/L, respectively (Fig. 1).

### Mediating role of BMI in associations of non-traditional CVRF with MetS

In this study, the mean BMI of those with MetS was 25.7 (3.3) kg/m^2^, significantly higher than that of those without MetS (*P* < 0.001). In the adjusted model, indirect associations via BMI implied a 28.7% (95%CI: 1.183–1.391) increase in MetS risk for HUA, a 10.4% (95%CI: 1.041–1.168) increase in MetS risk for HHCY, and a 12.5% (95%CI: 1.047–1.203) increase in MetS risk for HHsCRP. Proportions of associations of HUA, HHCY, and HHsCRP with MetS mediated by BMI were 43.89% (95%CI: 30.38–57.40%), 37.34% (95%CI: 13.86–60.83%), and 30.99% (95%CI: 13.16–48.83%), respectively. The adjusted mediation model at the continuous scale showed no substantial changes, except for Ln HCY OR(95%CI): 1.038(1.008–1.069), percentage mediated(%): 48.58(-6.03-103.20), (Table 3).

**Table 3:** Proportion of association of non-traditional CVRF with MetS mediated by BMI

	Unadjusted model		Adjusted model	
	Estimate(95%CI)	*P*-value	Estimate(95%CI)	*P*-value
Binary scale, HUA (vs. Non-HUA)				
Total Effect (OR)	1.609(1.295–1.923)	< 0.001	2.030(1.530–2.530)	< 0.001
Natural Direct Effect (OR)	1.324(1.079–1.569)	0.010	1.578(1.209–1.948)	0.002
Natural Indirect Effect (OR)	1.215(1.137–1.294)	< 0.001	1.287(1.183–1.391)	< 0.001
Percentage Mediated (%)	46.80(29.80–63.79)	< 0.001	43.89(30.38–57.40)	< 0.001
Continuous scale, Per SD in UA				
Total Effect (OR)	1.274(1.187–1.361)	< 0.001	1.485(1.352–1.618)	< 0.001
Natural Direct Effect (OR)	1.127(1.053–1.200)	< 0.001	1.268(1.159–1.377)	< 0.001
Natural Indirect Effect (OR)	1.131(1.103–1.159)	< 0.001	1.171(1.133–1.210)	< 0.001
Percentage Mediated (%)	53.76(39.56–67.96)	< 0.001	44.77(34.71–54.84)	< 0.001
Binary scale, HHCY (vs. Non-HHCY)				
Total Effect (OR)	1.023(0.885–1.160)	0.747	1.339(1.101–1.577)	0.005
Natural Direct Effect (OR)	0.975(0.851–1.099)	0.688	1.212(1.008–1.416)	0.042
Natural Indirect Effect (OR)	1.049(1.003–1.096)	0.038	1.104(1.041–1.168)	0.001
Percentage Mediated (%)	211.93(-997.27–1421.13)	0.731	37.34(13.86–60.83)	0.002
Continuous scale, Per SD in Ln HCY				
Total Effect (OR)	0.976(0.911–1.042)	0.479	1.082(0.984–1.179)	0.100
Natural Direct Effect (OR)	0.959(0.898–1.020)	0.191	1.042(0.953–1.131)	0.354
Natural Indirect Effect (OR)	1.018(0.995–1.040)	0.125	1.038(1.008–1.069)	0.015
Percentage Mediated (%)	-71.78(-319.92–176.36)	0.571	48.58(-6.03–103.20)	0.081
Binary scale, HHsCRP (vs. Non-HHsCRP)				
Total Effect (OR)	1.497(1.218–1.776)	0.0005	1.560(1.210–1.909)	0.002
Natural Direct Effect (OR)	1.318(1.085–1.552)	0.0076	1.386(1.090–1.682)	0.011
Natural Indirect Effect (OR)	1.136(1.069–1.203)	< 0.001	1.125(1.047–1.203)	0.002
Percentage Mediated (%)	36.02(18.78–53.25)	< 0.001	30.99(13.16–48.83)	0.001
Continuous scale, Per SD in Ln HsCRP				
Total Effect (OR)	1.360(1.266–1.455)	< 0.001	1.349(1.237–1.461)	< 0.001
Natural Direct Effect (OR)	1.213(1.132–1.293)	< 0.001	1.205(1.109–1.301)	< 0.001
Natural Indirect Effect (OR)	1.122(1.095–1.149)	< 0.001	1.120(1.088–1.152)	< 0.001
Percentage Mediated (%)	40.93(31.40–50.46)	< 0.001	41.34(29.58–53.09)	< 0.001

Note: Adjusted for age group, gender, occupation, education, monthly income, current smoking, current drinking, physical activity, ASCVD, liver, kidney, and thyroid dysfunction

Abbreviations: CVRF, cardiovascular risk factors; MetS, metabolic syndrome; BMI, body mass index; UA, uric acid; HUA, hyperuricemia; Ln HCY, natural logarithm of homocysteine; HHCY, hyperhomocysteinemia; Ln HsCRP, natural logarithm of hypersensitive C-reactive protein; HHsCRP, high hypersensitive C-reactive protein; ASCVD, arteriosclerotic cardiovascular disease; SD, standard deviation; OR, odds ratio; CI, confidence interval

**Table 4:** Joint association of non-traditional CVRF and BMI with MetS

		Unadjusted model		Adjusted model	
		OR(95%CI)	*P*-value	OR(95%CI)	*P*-value
Non-HUA	Underweight	0.527(0.338–0.822)	0.005	0.472(0.274–0.811)	0.007
	Normal weight	1.000 [ref.]		1.000 [ref.]	
	Overweight	2.768(2.385–3.212)	< 0.001	2.938(2.453–3.520)	< 0.001
	Obese	5.130(4.098–6.422)	< 0.001	4.931(3.765–6.457)	< 0.001
HUA	Underweight	4.243(1.324–13.595)	0.015	4.753(1.344–16.811)	0.016
	Normal weight	1.281(0.940–1.745)	0.117	1.392(0.956–2.026)	0.084
	Overweight	3.746(2.845–4.931)	< 0.001	5.860(4.059–8.461)	< 0.001
	Obese	6.001(3.892–9.252)	< 0.001	6.148(3.707–10.194)	< 0.001
Non-HHCY	Underweight	0.780(0.463–1.316)	0.352	0.690(0.362–1.314)	0.259
	Normal weight	1.000 [ref.]		1.000 [ref.]	
	Overweight	2.993(2.470–3.628)	< 0.001	3.014(2.398–3.789)	< 0.001
	Obese	5.366(4.024–7.155)	< 0.001	5.221(3.706–7.356)	< 0.001
HHCY	Underweight	0.594(0.325–1.086)	0.091	0.703(0.346–1.430)	0.331
	Normal weight	1.072(0.877–1.310)	0.498	1.258(0.977–1.620)	0.075
	Overweight	2.817(2.323–3.416)	< 0.001	3.989(3.107–5.121)	< 0.001
	Obese	5.235(3.929–6.975)	< 0.001	5.746(4.064–8.123)	< 0.001
Non-HHsCRP	Underweight	0.626(0.396–0.990)	0.045	0.595(0.338–1.047)	0.072
	Normal weight	1.000 [ref.]		1.000 [ref.]	
	Overweight	2.560(2.195–2.985)	< 0.001	2.931(2.429–3.537)	< 0.001
	Obese	4.592(3.657–5.767)	< 0.001	4.399(3.357–5.765)	< 0.001
HHsCRP	Underweight	1.067(0.377–3.015)	0.903	1.288(0.409–4.059)	0.665
	Normal weight	1.096(0.816–1.471)	0.542	1.271(0.901–1.793)	0.172
	Overweight	3.876(2.960–5.075)	< 0.001	4.026(2.906–5.580)	< 0.001
	Obese	6.578(4.257–10.162)	< 0.001	7.717(4.508–13.210)	< 0.001

Note: Adjusted for age group, gender, occupation, education, monthly income, current smoking, current drinking, physical activity, ASCVD, liver, kidney, and thyroid dysfunction

Considering ordinal BMI, interactions of abnormal status of non-traditional CVRF and BMI were more interested. Additive interaction RERI(95%CI): HUA + abnormal BMI: 2.521(0.793–4.250); HHCY + abnormal BMI: 0.589(-0.182-1.360); HHsCRP + abnormal BMI: 1.255(0.028–2.483). Multiplicative interaction OR(95%CI): HUA * abnormal BMI: 1.408(0.881–2.250); HHCY * abnormal BMI: 1.002(0.734–1.368); HHsCRP * abnormal BMI: 1.187(0.767–1.837)

Abbreviations: CVRF, cardiovascular risk factors; MetS, metabolic syndrome; BMI, body mass index; UA, uric acid; HUA, hyperuricemia; HCY, homocysteine; HHCY, hyperhomocysteinemia; HHsCRP, high hypersensitive C-reactive protein; ASCVD, arteriosclerotic cardiovascular disease; RERI, relative excess risk due to interaction; OR, odds ratio; CI, confidence interval

### Joint associations of non-traditional CVRF and BMI with MetS

A total of 1634 (37.5%), 1802 (41.3%), and 656 (15.0%) participants were normal weight, overweight, and obese, respectively. Abnormal non-traditional CVRF combined with overweight/obesity greatly increased MetS risk. Compared to non-HUA combined with normal weight, the adjusted ORs for MetS in HUA combined with overweight obesity were 5.860 (95%CI: 4.059–8.461) and 6.148 (95%CI: 3.707–10.194), respectively. Compared to non-HHCY combined with normal weight, the adjusted ORs for MetS in HHCY combined with overweight obesity were 3.989 (95%CI: 3.107–5.121) and 5.746 (95%CI: 4.064–8.123), respectively. Compared to non-HHsCRP combined with normal weight, the adjusted ORs for MetS in HHsCRP combined with overweight obesity were 4.026 (95%CI: 2.906–5.580) and 7.717 (95%CI: 4.508–13.210), respectively. Notably, normal weight might dilute significant effects of HUA, HHCY and HsCRP on MetS risk (adjusted OR(95%CI): HUA + normal weight: 1.392(0.956–2.026), HHCY + normal weight: 1.258(0.977–1.620), HHsCRP + normal weight: 1.271(0.901–1.793)) (Table 4).

Interactions of abnormal status of non-traditional CVRF and BMI were also evaluated to help further validate joint associations. HUA (RERI(95%CI): 2.521(0.793–4.250)) and HHsCRP (RERI(95%CI): 1.255(0.028–2.483)) showed significant additive interactions with abnormal BMI, indicating that MetS risk might be synergistically increased. HUA (OR(95%CI): 1.408(0.881–2.250)), HHCY (OR(95%CI): 1.002(0.734–1.368)), and HHsCRP (OR(95%CI): 1.187(0.767–1.837)) did not have any significant multiplicative interaction with abnormal BMI.

### Subgroup analyses

**Fig. 2:**
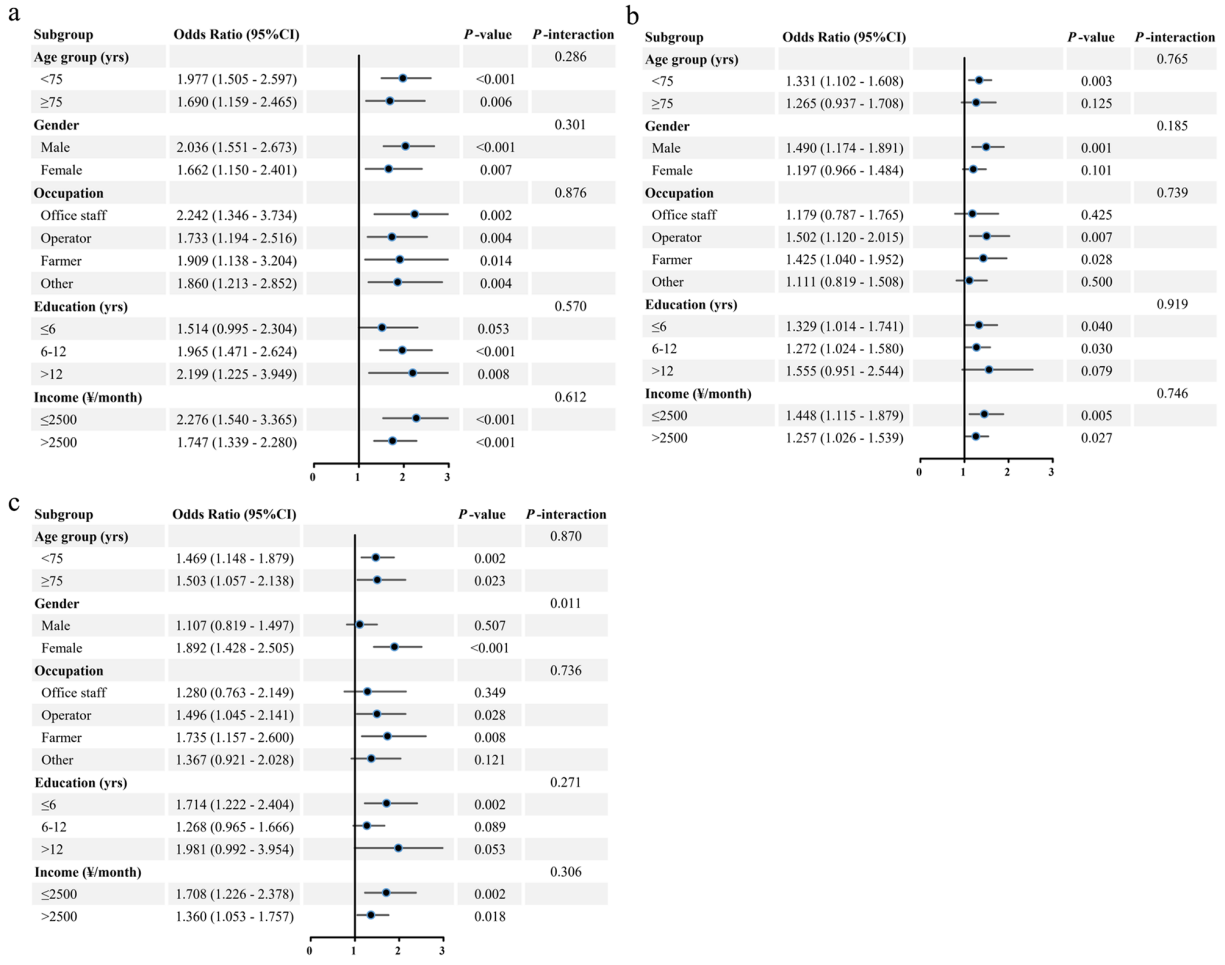
Association of non-traditional CVRF with MetS in demographic subgroups. **A: HUA; B: HHCY; C: HHsCRP.** After excluding specific demographic confounders included at the time of specific subgroup analysis, adjusted for age group, gender, occupation, education, monthly income, current smoking, current drinking, physical activity, ASCVD, liver, kidney, and thyroid dysfunction. Abbreviations: CVRF, cardiovascular risk factors; MetS, metabolic syndrome; HUA, hyperuricemia; HHCY, hyperhomocysteinemia; HHsCRP, high hypersensitive C-reactive protein; ASCVD, arteriosclerotic cardiovascular disease; CI, confidence interval

**Fig. 3:**
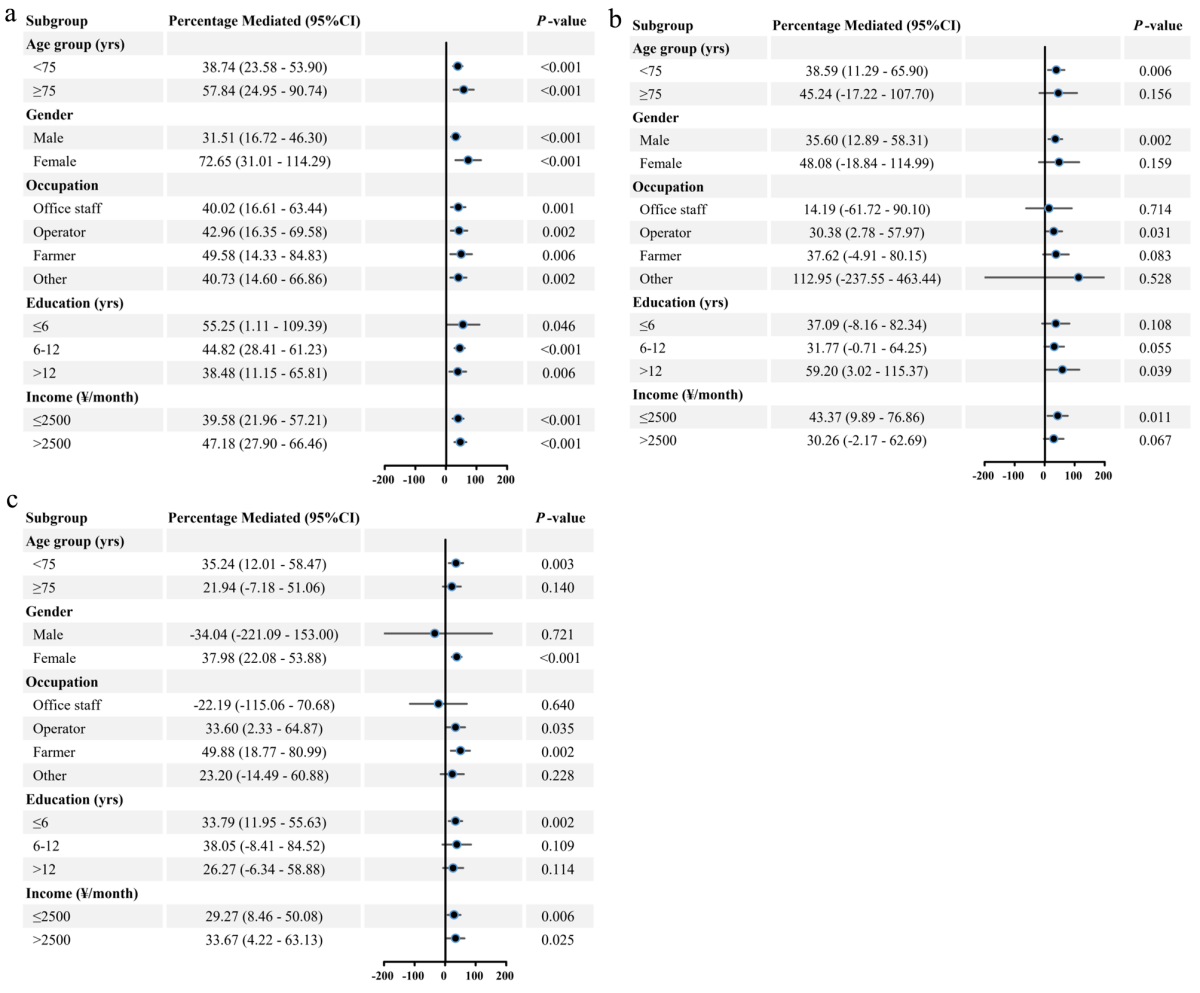
Proportion of association of non-traditional CVRF with MetS mediated by BMI in demographic subgroups. **A: HUA; B: HHCY; C: HHsCRP.** After excluding specific demographic confounders included at the time of specific subgroup analysis, adjusted for age group, gender, occupation, education, monthly income, current smoking, current drinking, physical activity, ASCVD, liver, kidney, and thyroid dysfunction. Abbreviations: CVRF, cardiovascular risk factors; MetS, metabolic syndrome; HUA, hyperuricemia; HHCY, hyperhomocysteinemia; HHsCRP, high hypersensitive C-reactive protein; ASCVD, arteriosclerotic cardiovascular disease; CI, confidence interval

Associations of UA with MetS were significantly stronger in participants aged < 75 years than in those aged ≥ 75 years, in females than in males, and in operators and farmers than in office staff at the continuous scale (*P*-interaction < 0.05) (Supplementary Fig. 2A). No statistical interaction was found for associations of HCY with MetS in demographic subgroups (*P*-interaction > 0.05), but stronger associations of HHCY with MetS were found in participants aged < 75 years (OR = 1.331, *P* = 0.003) than in those aged ≥ 75 years (OR = 1.265, *P* = 0.125), in males (OR = 1.490, *P* = 0.001) than in females (OR = 1.197, *P* = 0.101), and in operators (OR = 1.502, *P* = 0.007) and farmers (OR = 1.425, *P* = 0.028) than in office staff (OR = 1.179, *P* = 0.425) (Fig. 2B). Association of HsCRP with MetS was significantly stronger in females than in males at any scale (*P*-interaction < 0.05) (Fig. 2C and Supplementary Fig. 2C). Proportions of associations of non-traditional CVRF with MetS mediated by BMI in most demographic subgroups covered 95%CI of the main results (Fig. 3 and Supplementary Fig. 3). Joint associations of non-traditional CVRF and BMI with MetS in most demographic subgroups was also not obviously altered (Supplementary Fig. 4).

## Discussion

In this large, community-based study of the elderly population in China, the results indicated significant independent associations of HUA, HHCY and HHsCRP with MetS, in which BMI played moderate mediating roles. The higher risk of MetS was found in participants with abnormal non-traditional CRVF and overweight/obesity. The above findings were relatively consistent in demographic subgroups.

### Comparison of previous studies

The findings of associations between MetS and non-traditional CVRF represented by UA, HCY and HsCRP have previously been more controversial.

In terms of UA and MetS, the PREDIMED (Prevencion con Dieta Mediterranea) trial indicated that elevated UA was significantly associated with increased incidence of MetS in the European elderly population [53]. Nie et al. also found that UA was a predictor of MetS in the Chinese elderly population, independent of obesity [54]. The results of a meta-analysis by Yuan et al. indicated a positive dose-response relationship between UA and MetS risk [55]. However, several studies presented negative results, and clear differences between demographics. The PAMELA (Pressioni Arteriose Monitorate E Loro Associazioni) study indicated that UA was significantly associated with MetS after adjusting for age and gender, but this association disappeared after full adjustment [56]. It is speculated that (1) this may be due to overadjustment bias, where intermediate variable on the causal pathway from UA to MetS, i.e. MetS components, was adjusted for in the fully adjusted model, which may have weakened this association; and (2) this may be due to differences in study design and methodology, as the PAMELA study used a prospective design and modified Poisson regression to estimate relative risk (RR). The OR approximats the RR when the incidence is not common [57]. Studies from Taiwan, China found that the power of UA to predict MetS diminished with age, without clear relevance in females older than 75 years and in males older than 85 years [58, 59]. Ferrara et al. found that UA failed to predict MetS in an obese population [60], which might be due to the obscure effect of per unit change in UA on MetS and might therefore lead to the neglect of interventions for HUA to reduce MetS risk in an obese population. This study found a significant association between elevated UA and MetS risk, with BMI mediating about 40% of this association, and HUA combined with overweight/obesity had synergistic effects on MetS risk.

In terms of HCY and MetS. Hajer et al. found that HCY was significantly higher in MetS patients and increased with the presence of MetS components, but did not assess effect sizes [61]. Catena et al. found that elevated HCY was associated with MetS in hypertensive patients, but hypertension could be both a cause of MetS and a consequence of HHCY (H-type hypertension), thus limiting the interpretation of this finding [62]. Conversely, the PGHH (Persian Gulf Healthy Heart) study suggested no association between HCY and MetS in males (OR(95%CI): 1.00(0.98–1.02)) and females (OR(95%CI): 1.00(0.97–1.02)) [63], possibly because (1) less information on confounders was available, including only smoking, physical inactivity, insufficient fruit and vegetable intake, and BMI; and (2) a broader definition of MetS was used, excluding medication for hyperglycaemia and dyslipidaemia, which might have underestimated the prevalence of MetS. Considering the shortcomings of observational studies, Lee et al. conducted a Mendelian randomised study and found a causal link between HCY and increased risk of MetS in the Korean population [64]. In this study, HHCY, although not statistically different between MetS and non-MetS, significantly increased MetS risk after adjusting for confounders, especially in participants younger than 75 years and in males.

In terms of HsCRP and MetS, relatively few relevant studies have been conducted, especially involving BMI. Abu-Farha et al. found that in the Arab population, elevated HsCRP not only significantly increased MetS risk, but was also significantly associated with metabolic markers including BMI and insulin resistance [65]. This was consistent with the findings of Kawamoto et al. in the Japanese population [66]. Nevertheless, the results of Engelsen et al. suggested that the ability of HsCRP to predict MetS in the abdominally obese population was extremely limited (area under curve (95%CI): 0.57(0.53–0.60)) [67], but the results of Guven et al. suggested that abdominal obesity (OR(95%CI): 4.21(1.12–6.12)) was a key factor for elevated HsCRP in MetS patients [68]. Several studies have highlighted significant gender differences between HsCRP and MetS [69–71]. Garcia et al. found that HsCRP was significantly higher in females among MetS patients [70]. Both Han et al. and Hong et al. found a significant positive association between HsCRP and MetS only in females [69, 71]. The results of this study similarly displayed a significant gender interaction for this association, with females being higher than males. Considering that all females in this study were 60 years of age and older, after excluding the role of estrogen in the inflammatory process [72], possible reasons for this are (1) noting that this study included more females and more females had MetS, so more statistical power to identify meaningful results in females; and (2) females might have more systemic adipose tissue compared to males, which could be a source of pro-inflammatory cytokines [73]. Interestingly, BMI was found to play a moderate mediating role between HHsCRP and MetS in this study, and this mediating effect was significant only in females, which might help to corroborate the above reasons.

The current focus on associations of non-traditional CVRF, BMI with MetS has not been reported, mostly focusing on those between the two in a specific population. This study provides an opportunity to quantify how BMI affects traditional and non-traditional CVRF, with BMI acting as the stronger mediator and synergist between traditional and non-traditional CVRF. This might be a potential signal to further explore the mechanisms of BMI in cardiometabolic disease and strengthen the position of weight management in fighting cardiometabolic disease.

### Potential mechanisms

Although mechanisms linking UA, HCY and HsCRP to MetS have not been fully understood, it is hypothesized that these might be related to oxidative stress and inflammation [74–76]. BMI, a measure of general obesity, was considered a marker of systemic inflammation due to its close association with pro-inflammatory cytokines, which further reinforced possible mechanisms between non-traditional CVRF and MetS [77]. Also, non-traditional CVRF and BMI formed a mutually reinforcing vicious circle [78–80].

Elevated UA, a marker of oxidative stress, could lead to increased production of reactive oxygen species, which could damage cells and tissues in the body and induce inflammation [74]. This inflammation could promote insulin resistance to increase MetS risk. Elevated UA might also lead to changes in BP and lipid metabolism through endothelial dysfunction [81], which could further promote MetS.

Elevated HCY, a sulfur-containing amino acid, could increase MetS risk through MetS components. First, elevated HCY could lead to a decrease in nitric oxide, a molecule that helps regulate BP, so elevated HCY was associated with an increased risk of hypertension [82]. Second, elevated HCY could either impair insulin secretion through alterations in beta-cell glucose metabolism and generation of key stimulus-secretion coupling factors [83], or induce insulin resistance and cause diabetic phenotypes by protein cysteine-homocysteinylation of the pro-insulin receptor [25]. Finally, elevated HCY could impair the body’s ability to produce and use HDL-C, thereby increase the risk of dyslipidemia [84].

Elevated HsCRP, a marker of inflammation, is produced in response to inflammation [44]. Elevated HsCRP could be involved in MetS by promoting insulin resistance, increasing oxidative stress and inflammation, and contributing to dyslipidemia [76]. HsCRP could inhibit adiponectin activity, an important hormone that helps regulate metabolism and is associated with MetS [85]. HsCRP has also been associated with changes in gut microbiome, which could lead to metabolic dysfunction and thus cause MetS [86].

## Strengths and limitations

Strengths of this study include the large naturally elderly population (4360 analysis sample), the high degree of participant cooperation (91.7%), the availability of self-reported information and measurement factors for risk estimation, and the retrospective validation of medical records for disease and medication history. Additionally, relatively comprehensive analytical approaches, multivariate adjustment strategies, and various scales of relevant variables were used to test the reliability of the resulting associations.

Limitations of this study equally could not be ignored. First, due to the observational design, causal inferences could not be made for associations of non-traditional CVRF and BMI with MetS, particularly the mediating role played by the BMI change trajectory. Second, despite model adjustment for most covariates, residual confounders were unavoidable, particularly because nutritional food intake effectively altered HUA, HHCY, and inflammation. In randomized controlled trials of folic acid, vitamin B6 and vitamin B12 supplements involved in HCY metabolism, no clinically significant benefits were found, so the veracity of these associations is questionable [87]. Third, the generalisability of the findings to other characterised populations was limited by the homogeneity of the participants in this study, i.e. poorly educated, predominantly physically active, and mostly hypertensive. Finally, due to the nature of post hoc subgroup analyses, sample sizes for individual subgroups were not calculated prior to data collection, which might lead to undercounting of participants and events, and therefore results should be interpreted cautiously.

## Conclusions

High prevalence of MetS and HHCY, and relatively low prevalence of HUA and HHsCRP were observed in the Chinese elderly population. HUA, HHCY, and HHsCRP were all significantly and independently associated with MetS, and these associations remained stable in most demographic subgroups. This supports the potential of focusing on non-traditional CVRF interventions for preventing and controlling MetS. BMI played moderate mediating roles in associations between non-traditional CVRF and MetS, hinting at the clinical benefit of weight loss for reducing MetS risk in non-traditional CVRF interventions. The higher risk of MetS was found in participants with abnormal non-traditional CVRF and overweight/obesity, but MetS risk was not significant when weight was normal, even with abnormal non-traditional CVRF. This highlights the protection of maintaining normal weight to regulate CVRF. The above findings warrant further exploration in prospective studies.

## Electronic supplementary material

Below is the link to the electronic supplementary material.

Supplementary Material 1

## Data Availability

The data that support the findings of this study are available from the corresponding author upon reasonable request.

## Abbreviations

ALT: alanine aminotransferase;
AST: aspartate aminotransferase;
ASCVD: arteriosclerotic cardiovascular disease;
BMI: body mass index;
CI: confidence interval;
CVRF: cardiovascular risk factors;
DBP: diastolic blood pressure;
FPG: fasting plasma glucose;
GFR: glomerular filtration rate;
HCY: homocysteine;
HDL-C: high-density lipid cholesterol;
HHCY: hyperhomocysteinemia;
HHsCRP: high hypersensitive C-reactive protein;
HUA: hyperuricemia;
IQR: interquartile range;
LDL-C: low-density lipid cholesterol;
Ln: natural logarithm;
MetS: metabolic syndrome;
OR: odds ratio;
RERI: relative excess risk due to interaction;
SBP: systolic blood pressure;
SD: standard deviation;
SHECH: Shanghai Elderly Cardiovascular Health;
TC: total cholesterol;
TG: triglyceride;
TSH: thyroid stimulating hormone;
UA: uric acid;
WC: waist circumference
