# Supplementary material for: Associations of non-traditional cardiovascular risk factors and body mass index with metabolic syndrome in the Chinese elderly population
Source: Diabetol Metab Syndr. 2023 Jun 16;15:129. doi: 10.1186/s13098-023-01047-4 (PMC10273701; doi:10.1186/s13098-023-01047-4)
Supplement: Supplementary file 1 — Supplementary Material 1 [file 13098_2023_1047_MOESM1_ESM.docx]

Supplementary Table 1. Spearman's rank correlation coefficients of non-traditional CVRF and BMI with MetS.

|  | UA | | HCY | | HsCRP | | BMI | |
| --- | --- | --- | --- | --- | --- | --- | --- | --- |
|  | ***ρ*** | *P*-value | ***ρ*** | *P*-value | ***ρ*** | *P*-value | ***ρ*** | *P*-value |
| WC | 0.171 | <0.001 | 0.118 | <0.001 | 0.183 | <0.001 | 0.587 | <0.001 |
| FPG | 0.005 | 0.756 | -0.014 | 0.340 | 0.085 | <0.001 | 0.200 | <0.001 |
| SBP | 0.050 | 0.001 | 0.083 | <0.001 | 0.069 | <0.001 | 0.155 | <0.001 |
| DBP | 0.060 | <0.001 | -0.015 | 0.321 | 0.038 | 0.017 | 0.135 | <0.001 |
| MAP | 0.064 | <0.001 | 0.038 | 0.014 | 0.066 | <0.001 | 0.171 | <0.001 |
| TG | 0.206 | <0.001 | -0.009 | 0.545 | 0.172 | <0.001 | 0.242 | <0.001 |
| HDL-C | -0.275 | <0.001 | -0.155 | <0.001 | -0.217 | <0.001 | -0.288 | <0.001 |
| TG/HDL-C | 0.259 | <0.001 | 0.056 | <0.001 | 0.215 | <0.001 | 0.292 | <0.001 |
| Standardized score of MetS | 0.160 | <0.001 | 0.081 | <0.001 | 0.220 | <0.001 | 0.465 | <0.001 |
| Number of MetS components | 0.141 | <0.001 | -0.008 | 0.670 | 0.192 | <0.001 | 0.407 | <0.001 |

Note: MAP is the sum of one-third of SBP in mmHg and two-thirds of DBP in mmHg; TG/HDL-C is the ratio of TG in mg/dL to HDL-C in mg/dL; Standardized score of MetS is the sum of standardized scores of WC, FPG, MAP and TG/HDL-C. Number of MetS components ranges from 0 to 5.

Abbreviations: CVRF, cardiovascular risk factors; BMI, body mass index; MetS, metabolic syndrome; UA, uric acid; HCY, homocysteine; HsCRP, hypersensitive C-reactive protein; WC, waist circumference; FPG; fasting plasma glucose; SBP, systolic blood pressure; DBP, diastolic blood pressure; MAP, mean arterial pressure; TG, triglyceride; HDL-C, high-density lipid cholesterol.

Supplementary Figure 1. Study flow.

Abbreviations: SHECH, Shanghai Elderly Cardiovascular Health; MetS, metabolic syndrome.

**
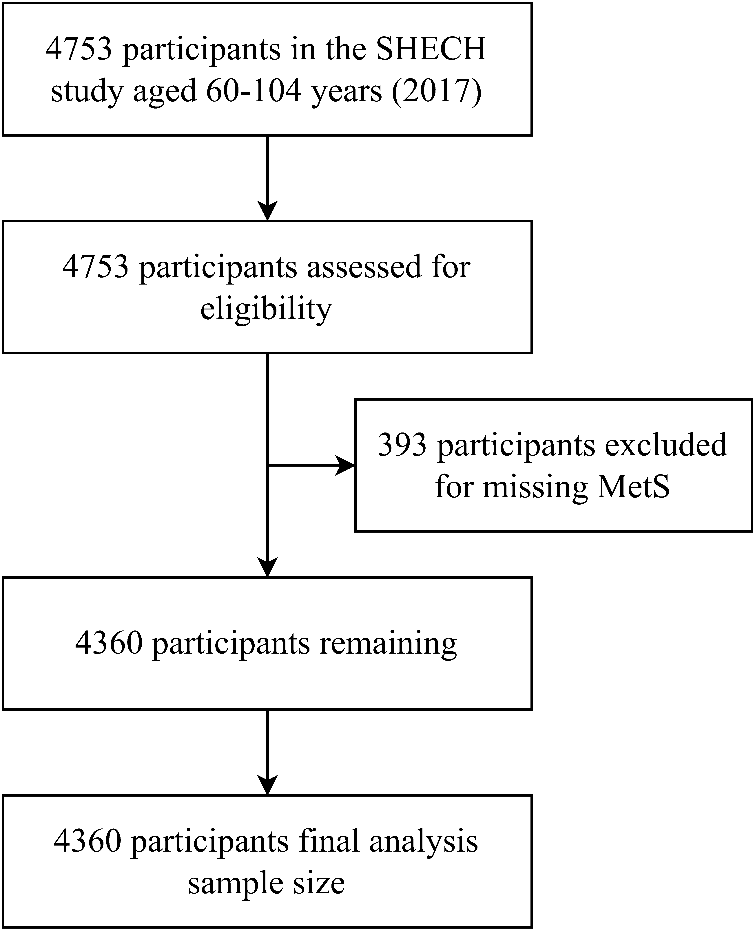
**

Supplementary Figure 2. Association of non-traditional CVRF with MetS in demographic subgroups. **A: Per SD in UA; B: Per SD in Ln HCY; C: Per SD in Ln HsCRP.** After excluding specific demographic confounders included at the time of specific subgroup analysis, adjusted for age group, gender, occupation, education, monthly income, current smoking, current drinking, physical activity, ASCVD, liver, kidney, and thyroid dysfunction.

Abbreviations: CVRF, cardiovascular risk factors; MetS, metabolic syndrome; UA, uric acid; Ln HCY, natural logarithm of homocysteine; Ln HsCRP, natural logarithm of hypersensitive C-reactive protein; ASCVD, arteriosclerotic cardiovascular disease; SD, standard deviation; CI, confidence interval.

**A**

**
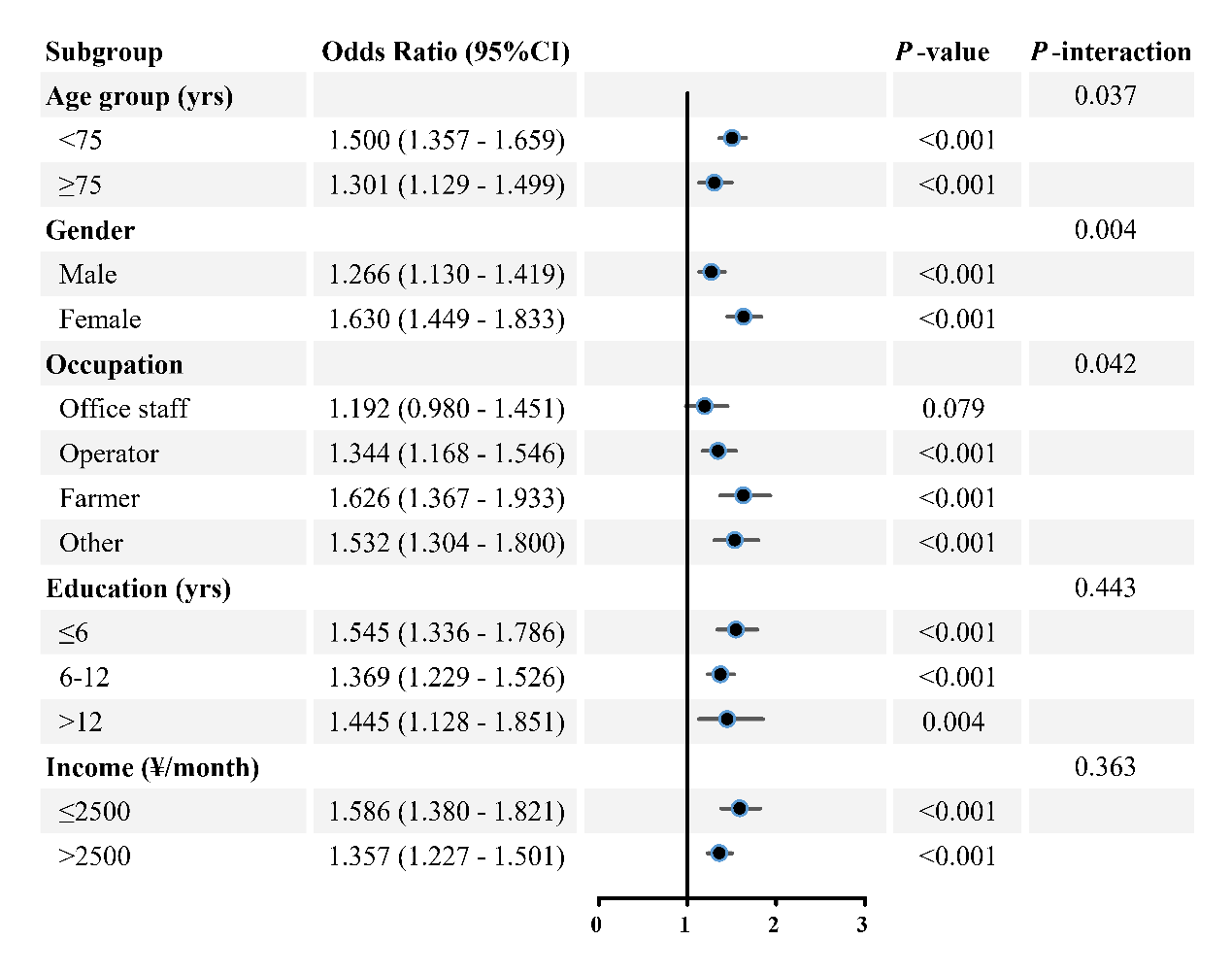
**

**B**

**
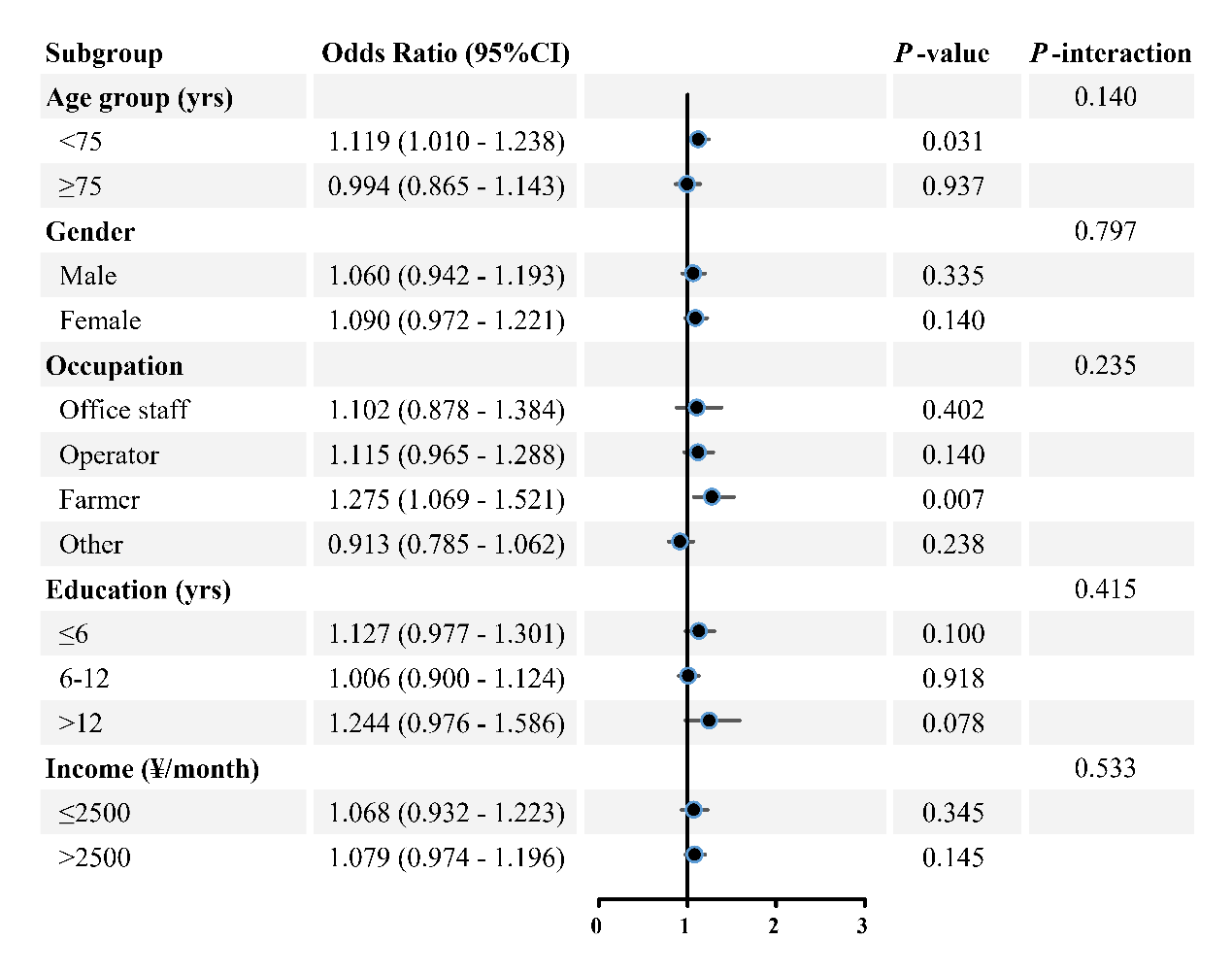
**

**C**

**
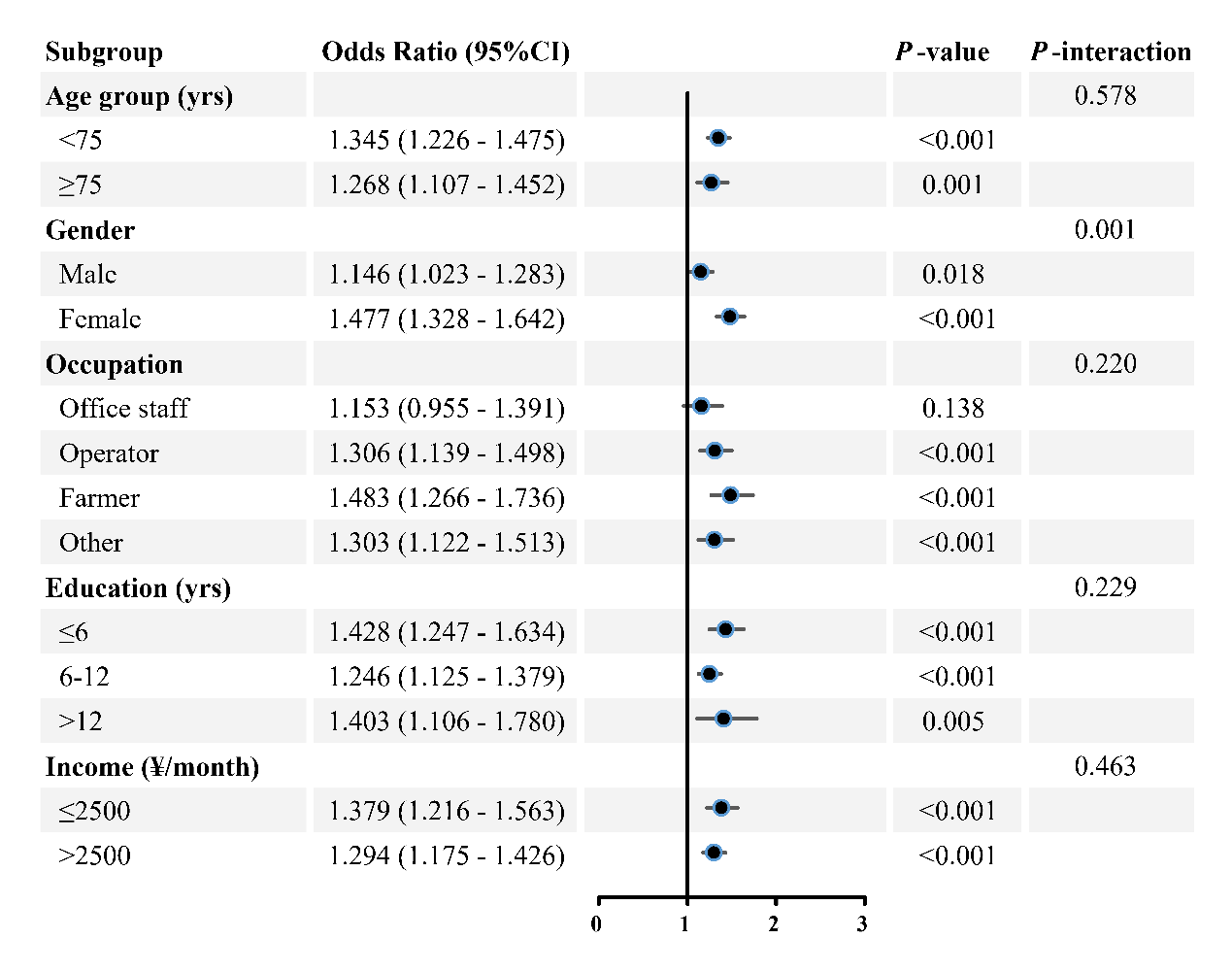
**

Supplementary Figure 3. Association of non-traditional CVRF with MetS in demographic subgroups. **A: Per SD in UA; B: Per SD in Ln HCY; C: Per SD in Ln HsCRP.** After excluding specific demographic confounders included at the time of specific subgroup analysis, adjusted for age group, gender, occupation, education, monthly income, current smoking, current drinking, physical activity, ASCVD, liver, kidney, and thyroid dysfunction.

Abbreviations: CVRF, cardiovascular risk factors; MetS, metabolic syndrome; UA, uric acid; Ln HCY, natural logarithm of homocysteine; Ln HsCRP, natural logarithm of hypersensitive C-reactive protein; ASCVD, arteriosclerotic cardiovascular disease; SD, standard deviation; CI, confidence interval.

**A**

**
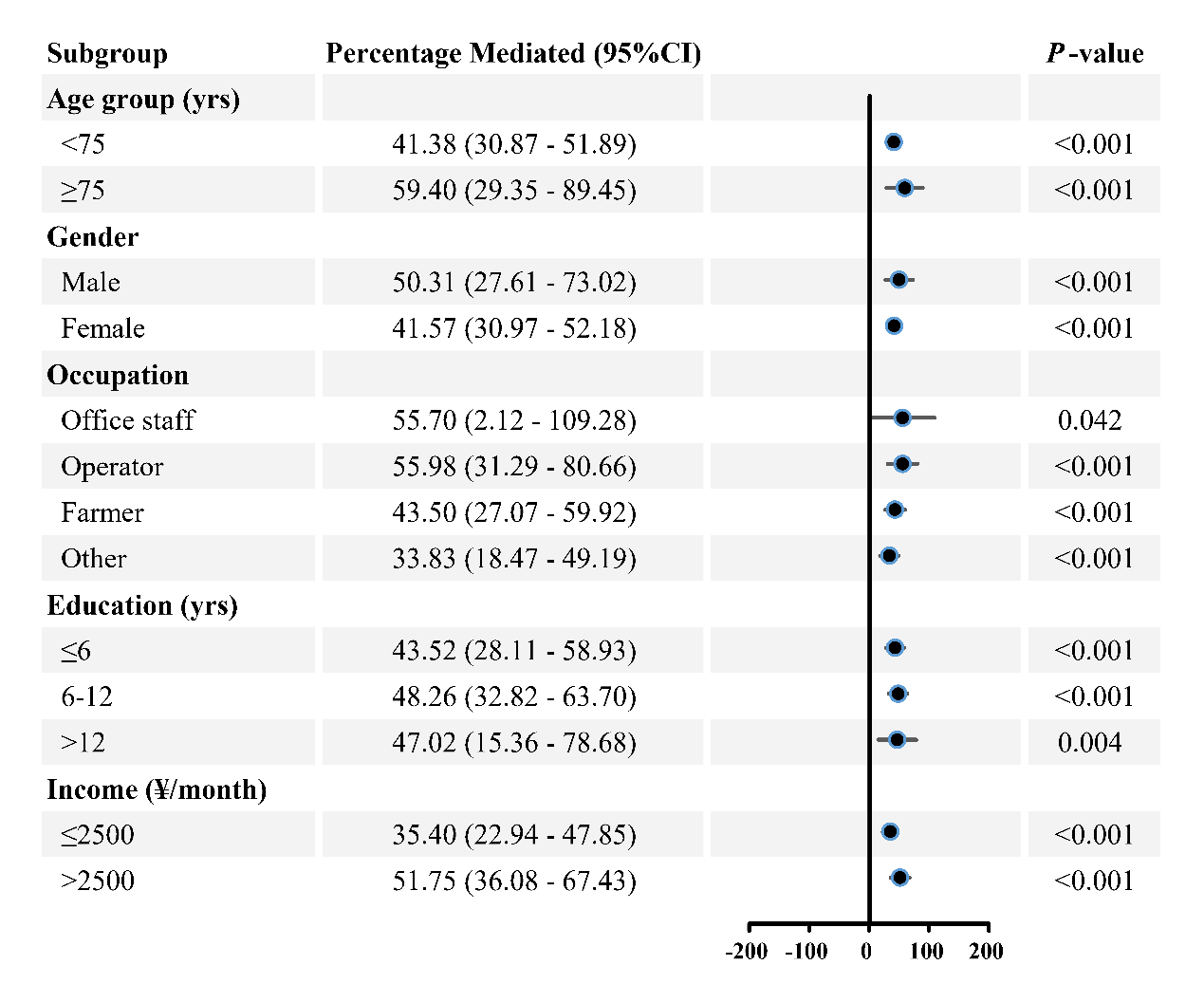
**

**B**

**
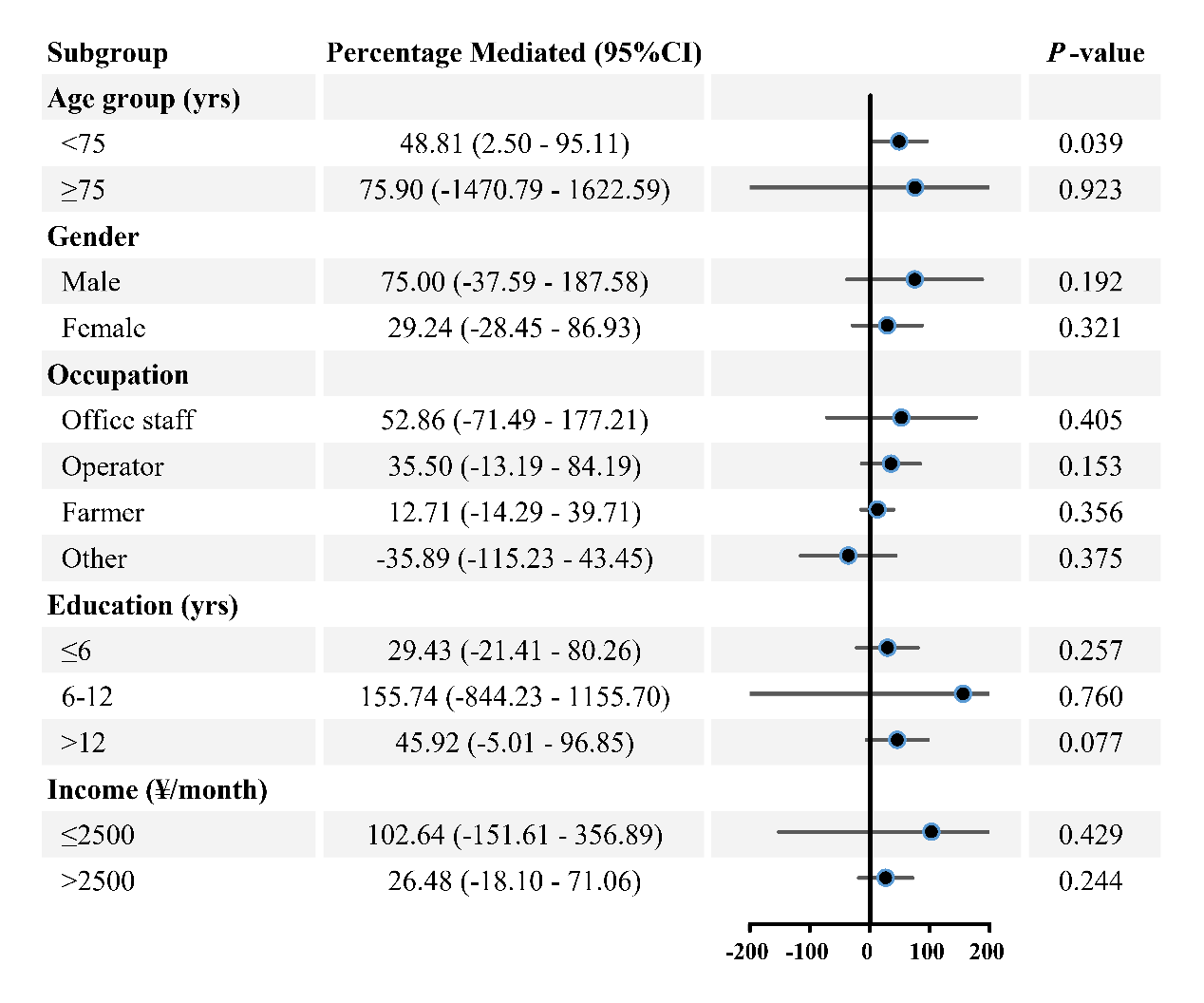
**

**C**

**
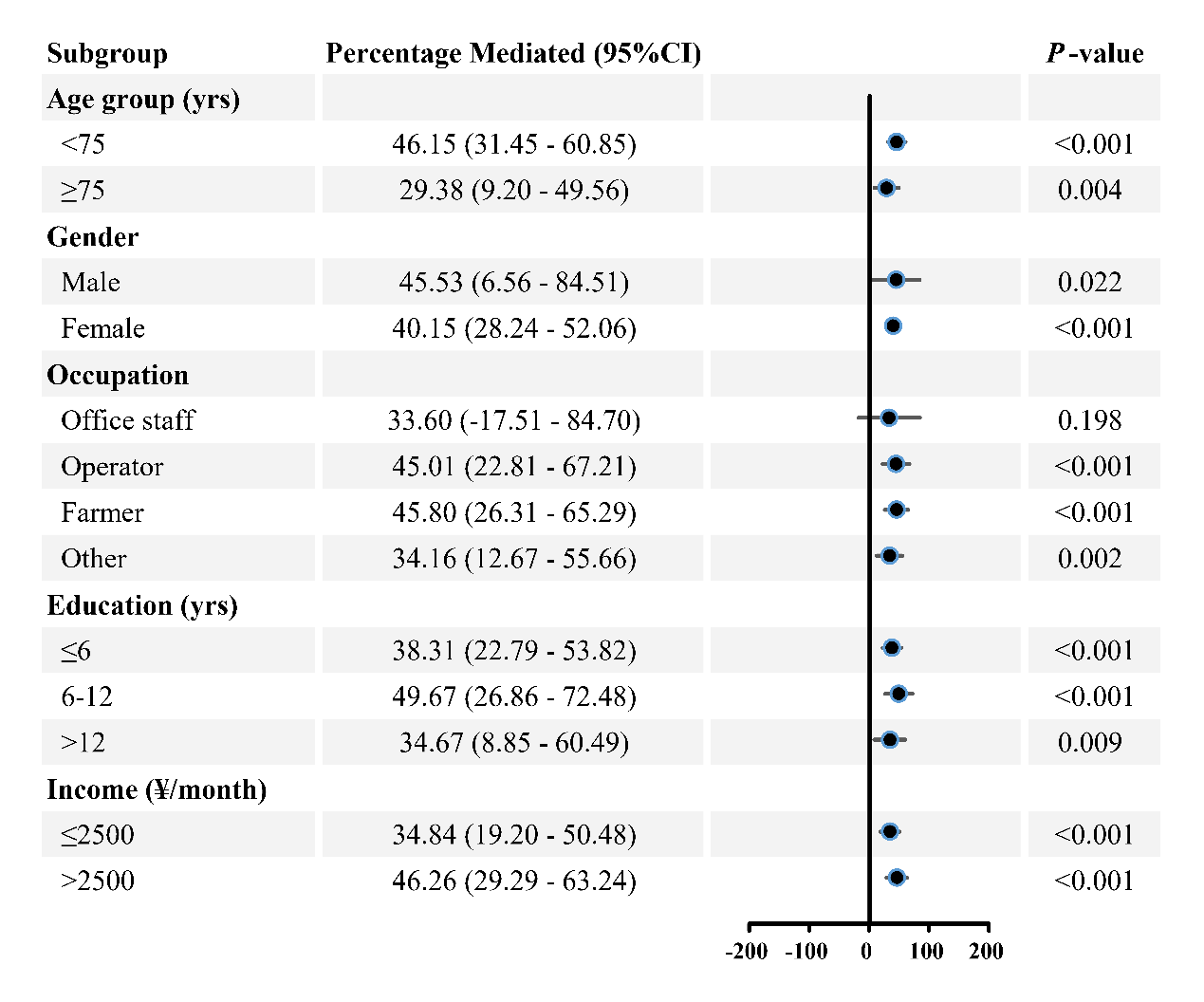
**

Supplementary Figure 4. Joint association of non-traditional CVRF and BMI with MetS in demographic subgroups. **A.1: Age group (UA and BMI); A.2: Age group (HCY and BMI); A.3: Age group (HsCRP and BMI); B.1: Gender (UA and BMI); B.2: Gender (HCY and BMI); B.3: Gender (HsCRP and BMI); C.1: Occupation (UA and BMI); C.2: Occupation (HCY and BMI); C.3: Occupation (HsCRP and BMI); D.1: Education (UA and BMI); D.2: Education (HCY and BMI); D.3: Education (HsCRP and BMI); E.1: Monthly income (UA and BMI); E.2: Monthly income (HCY and BMI); E.3: Monthly income (HsCRP and BMI).** After excluding specific demographic confounders included at the time of specific subgroup analysis, adjusted for age group, gender, occupation, education, monthly income, current smoking, current drinking, physical activity, ASCVD, liver, kidney, and thyroid dysfunction.

Abbreviations: CVRF, cardiovascular risk factors; MetS, metabolic syndrome; BMI, body mass index; UA, uric acid; HUA, hyperuricemia; HCY, homocysteine; HHCY, hyperhomocysteinemia; HHsCRP, high hypersensitive C-reactive protein; ASCVD, arteriosclerotic cardiovascular disease; CI, confidence interval.

**A.1**

**
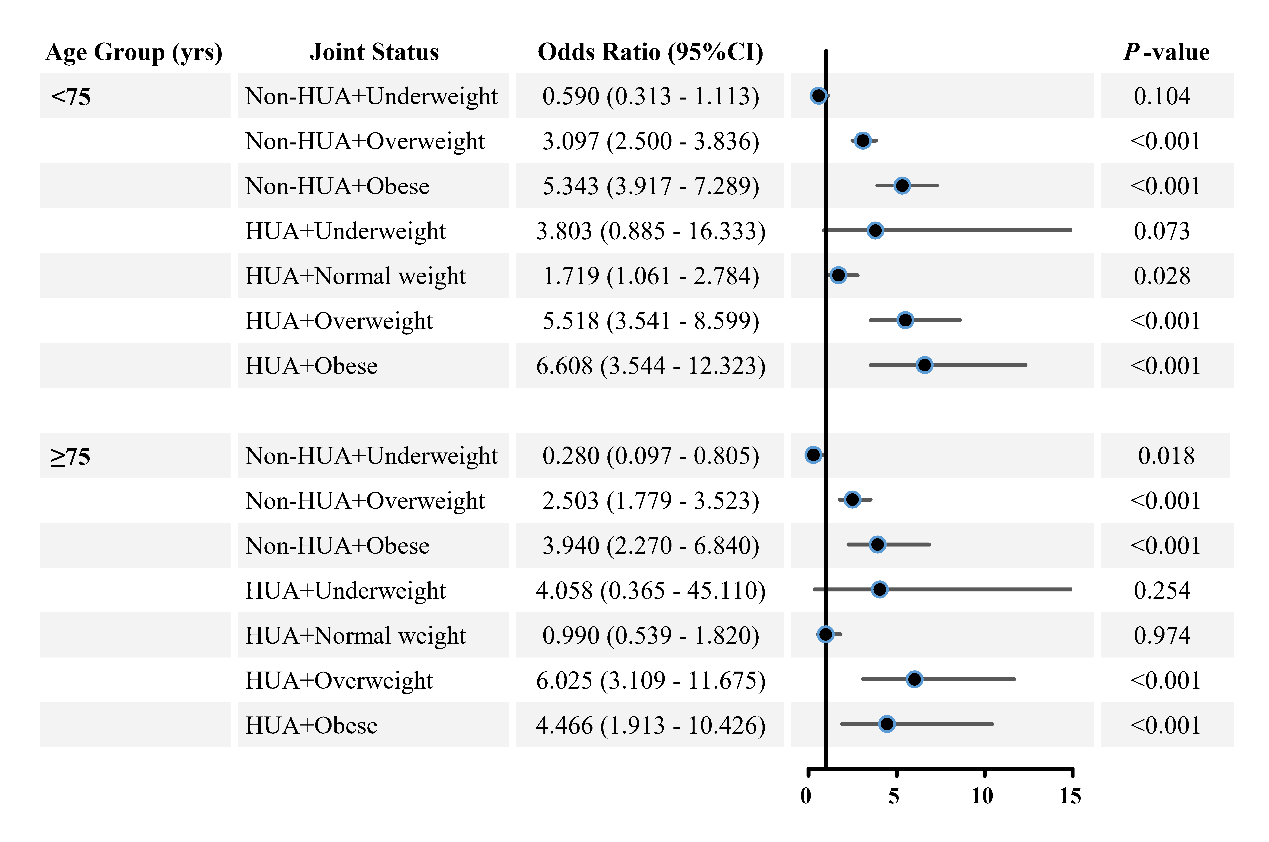
**

**A.2**

**
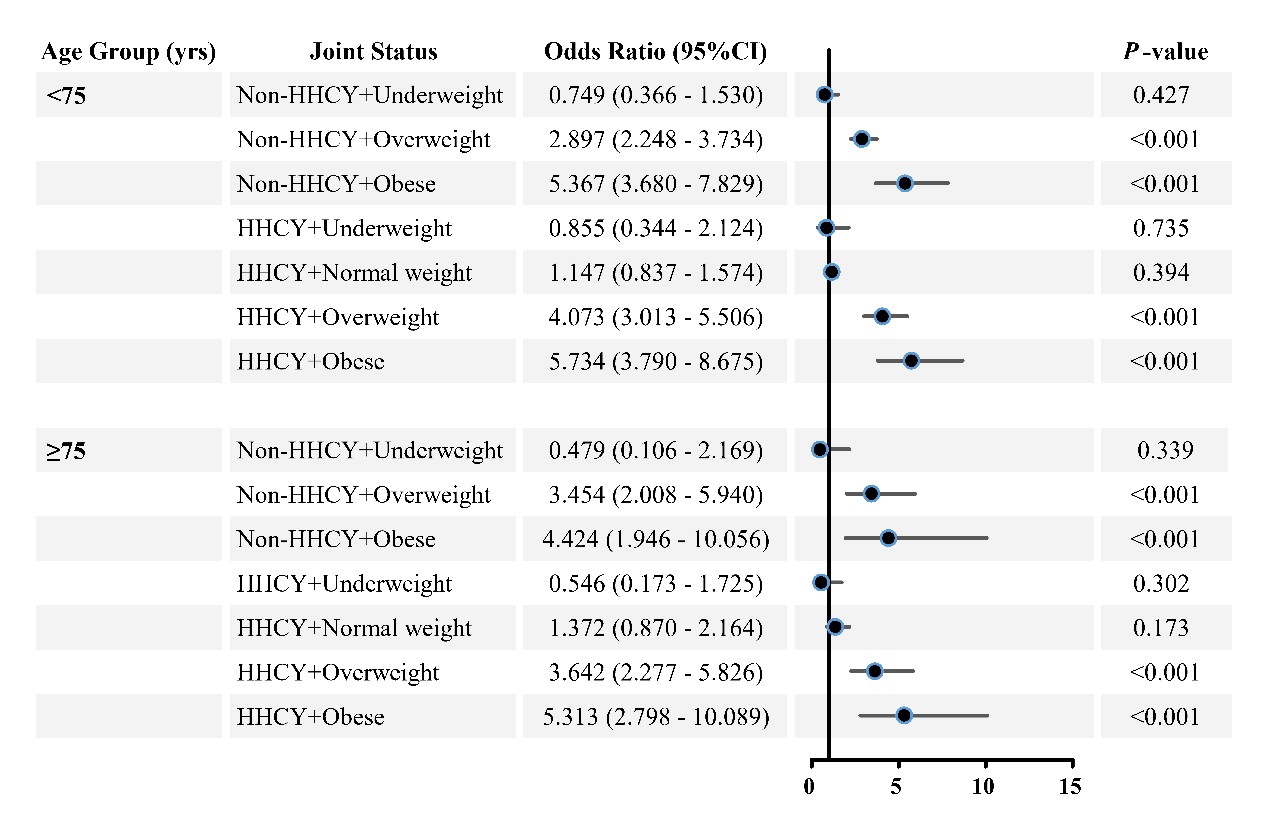
**

**A.3**

**
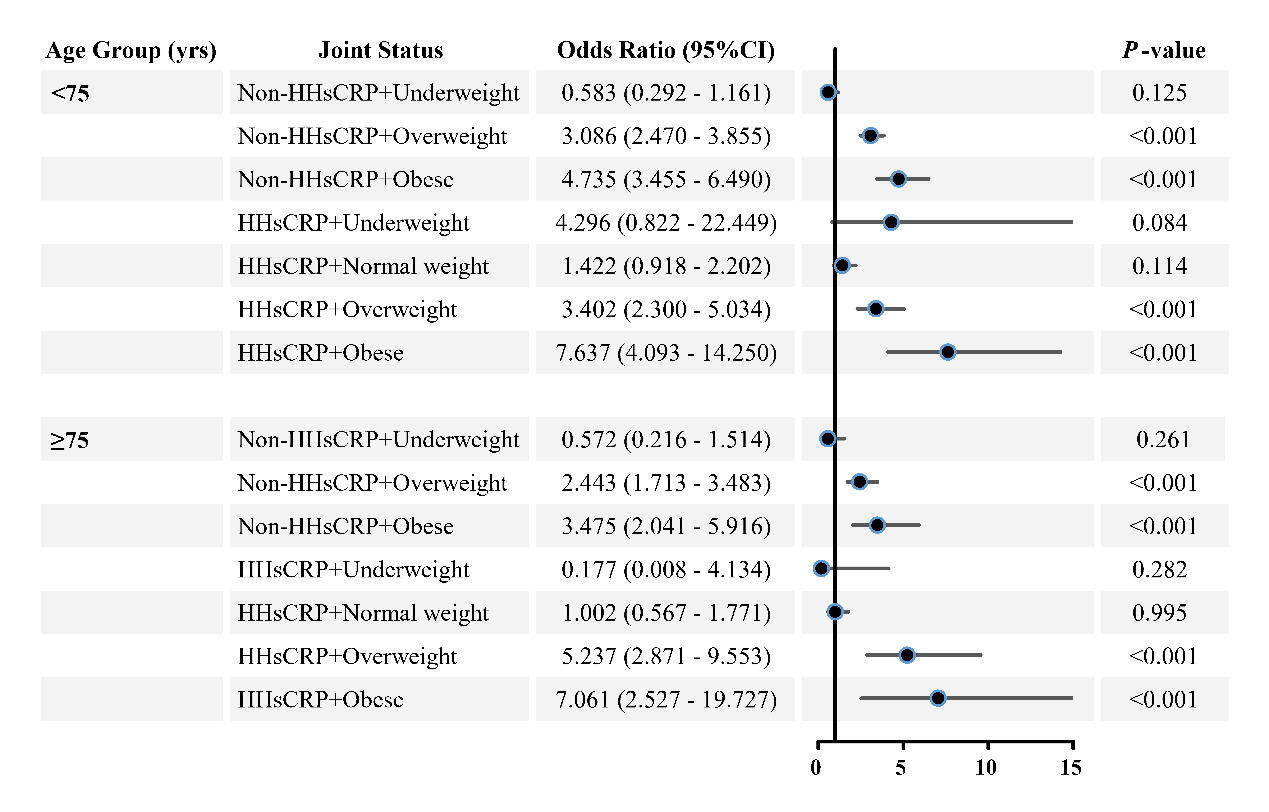
**

**B.1**

**
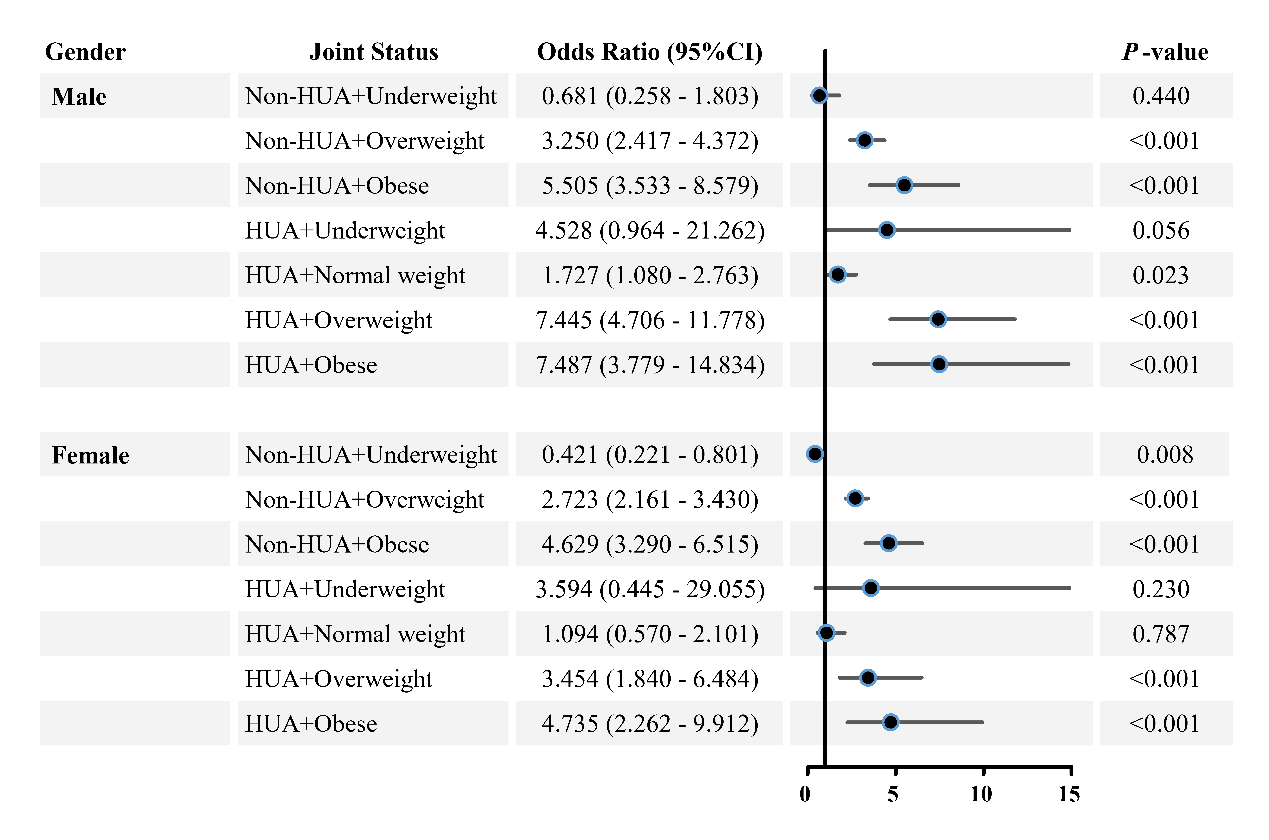
**

**B.2**

**
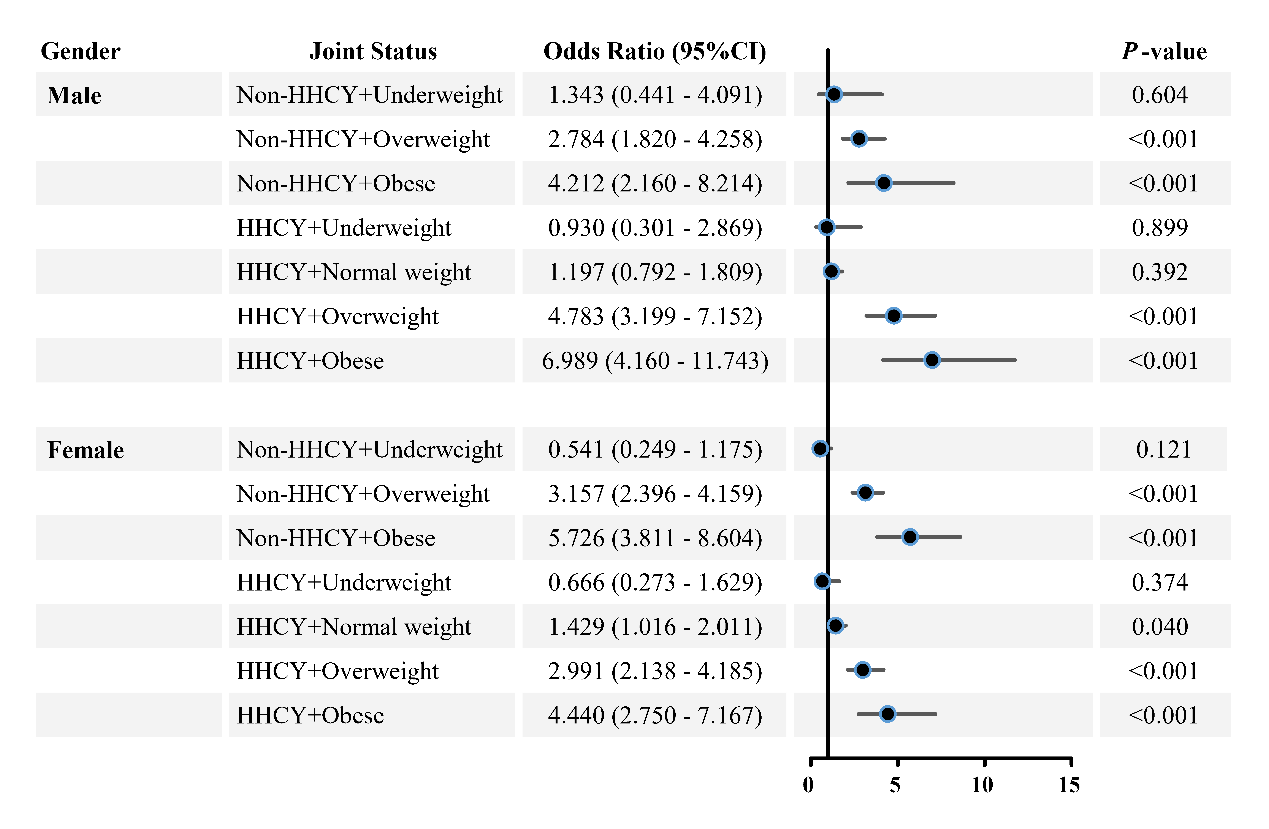
**

**B.3**

**
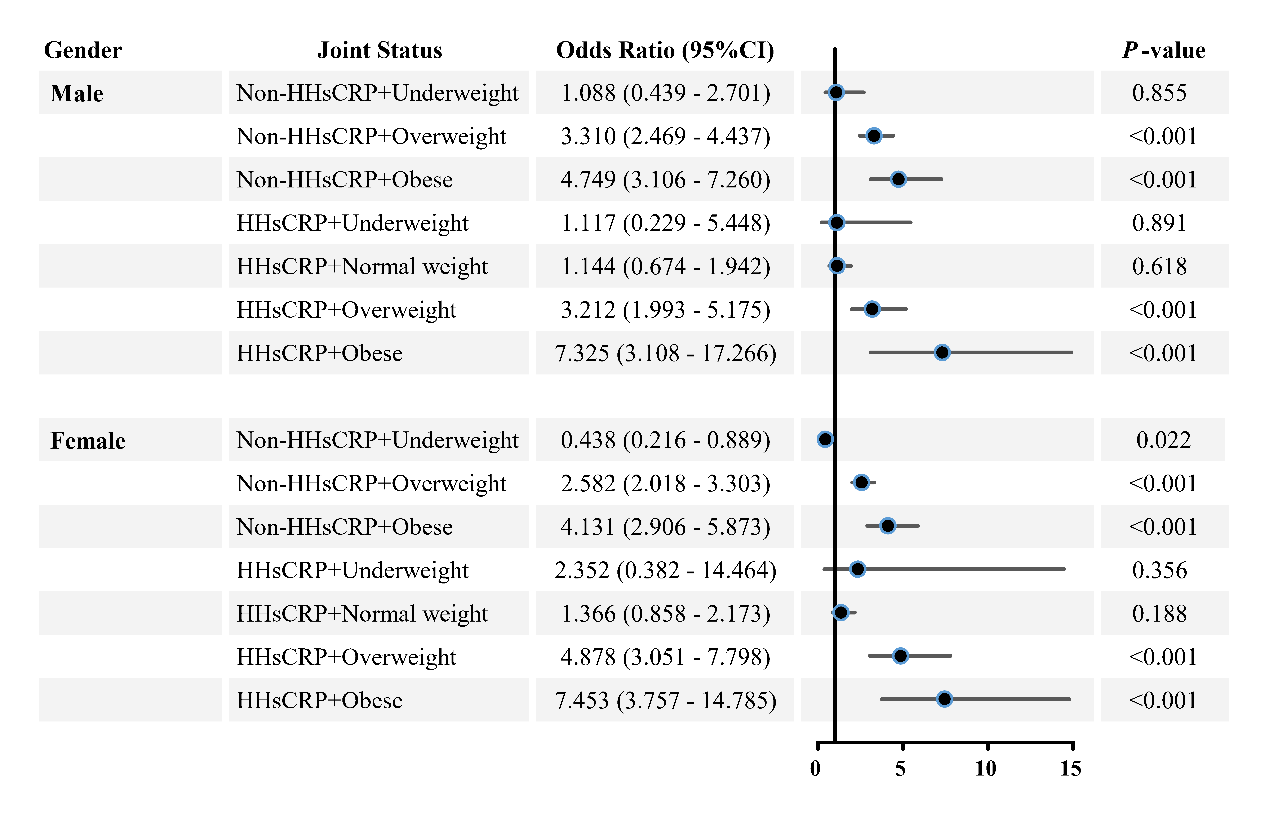
**

**C.1**

**
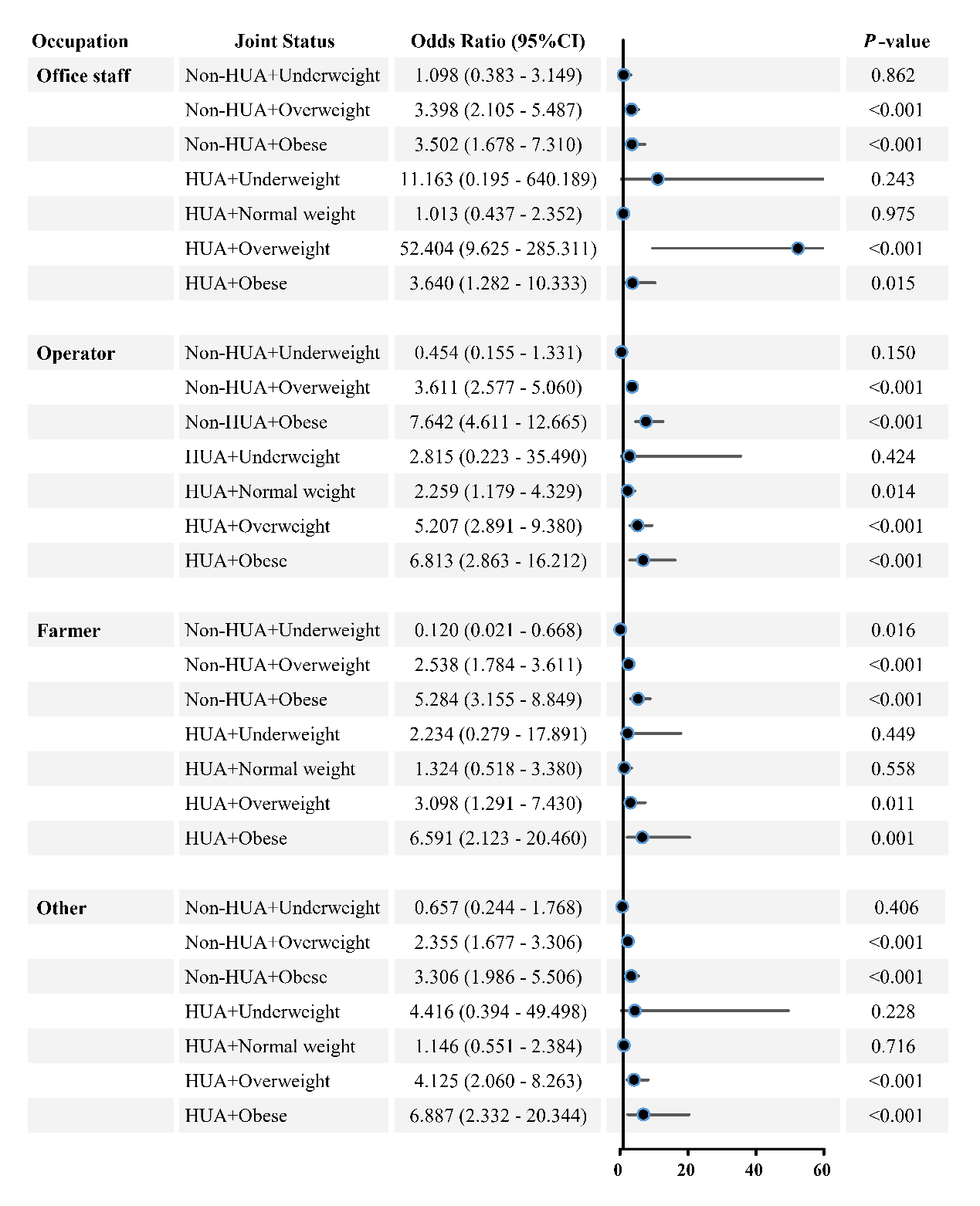
**

**C.2**

**
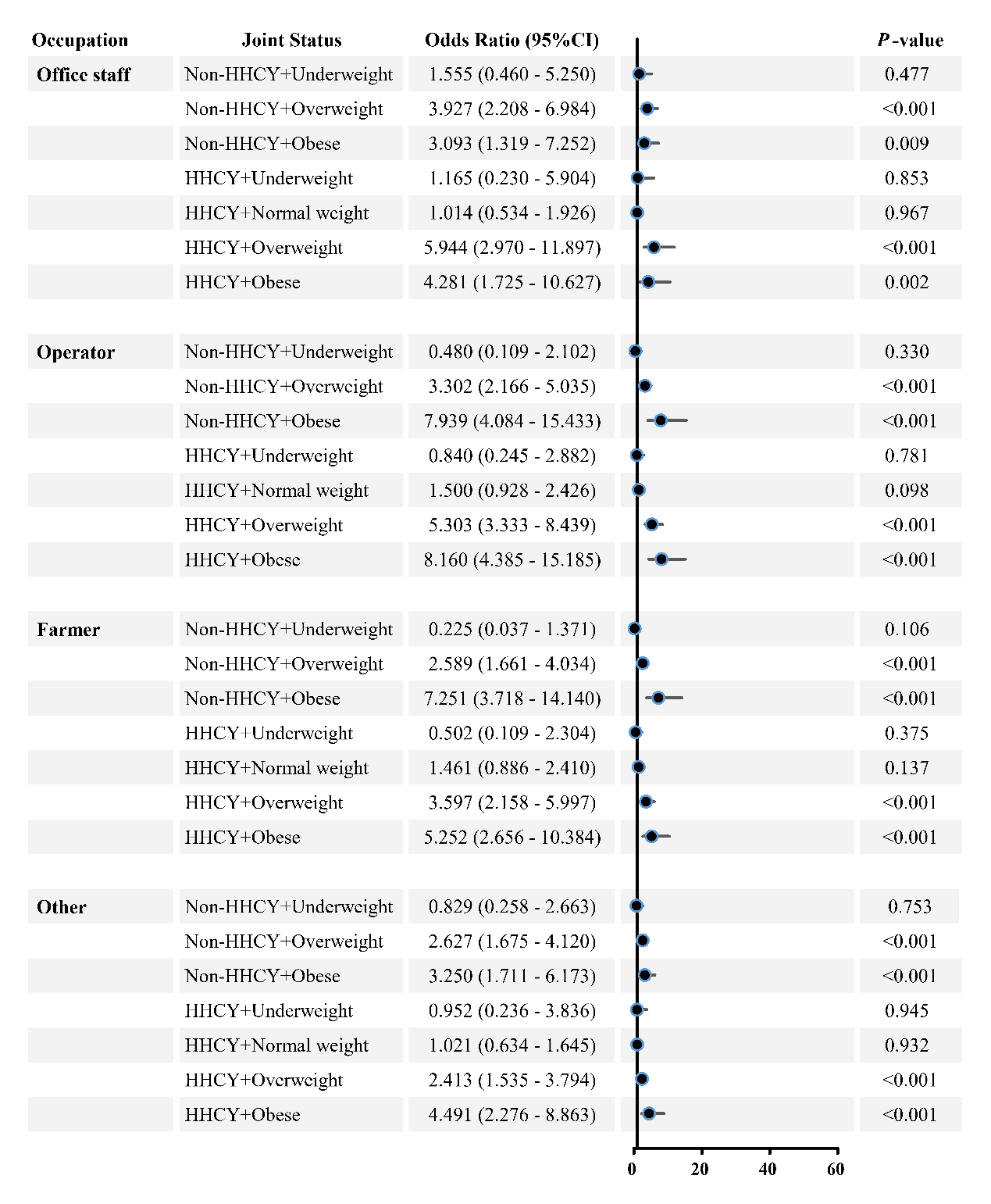
**

**C.3**

**
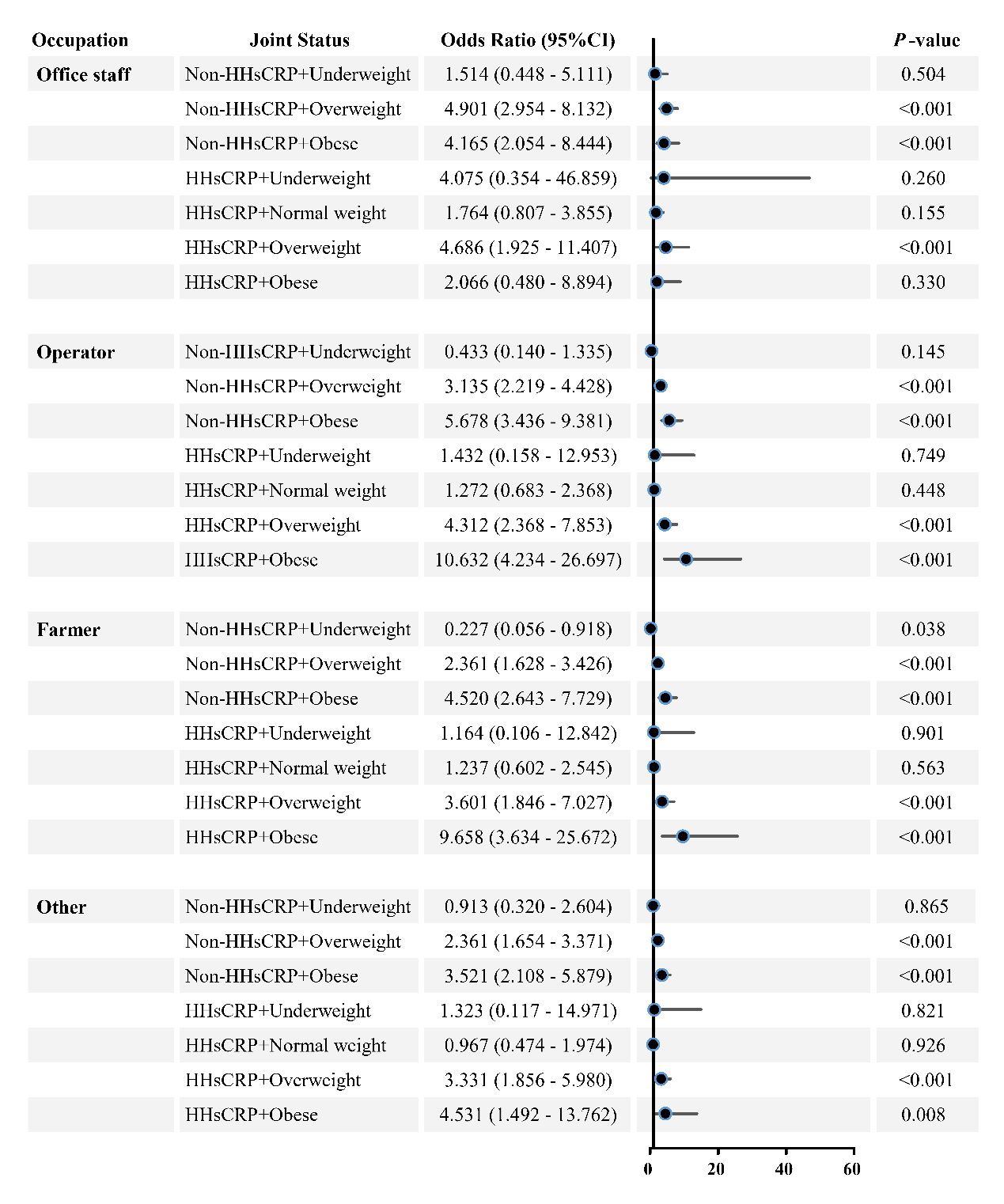
**

**D.1**

**
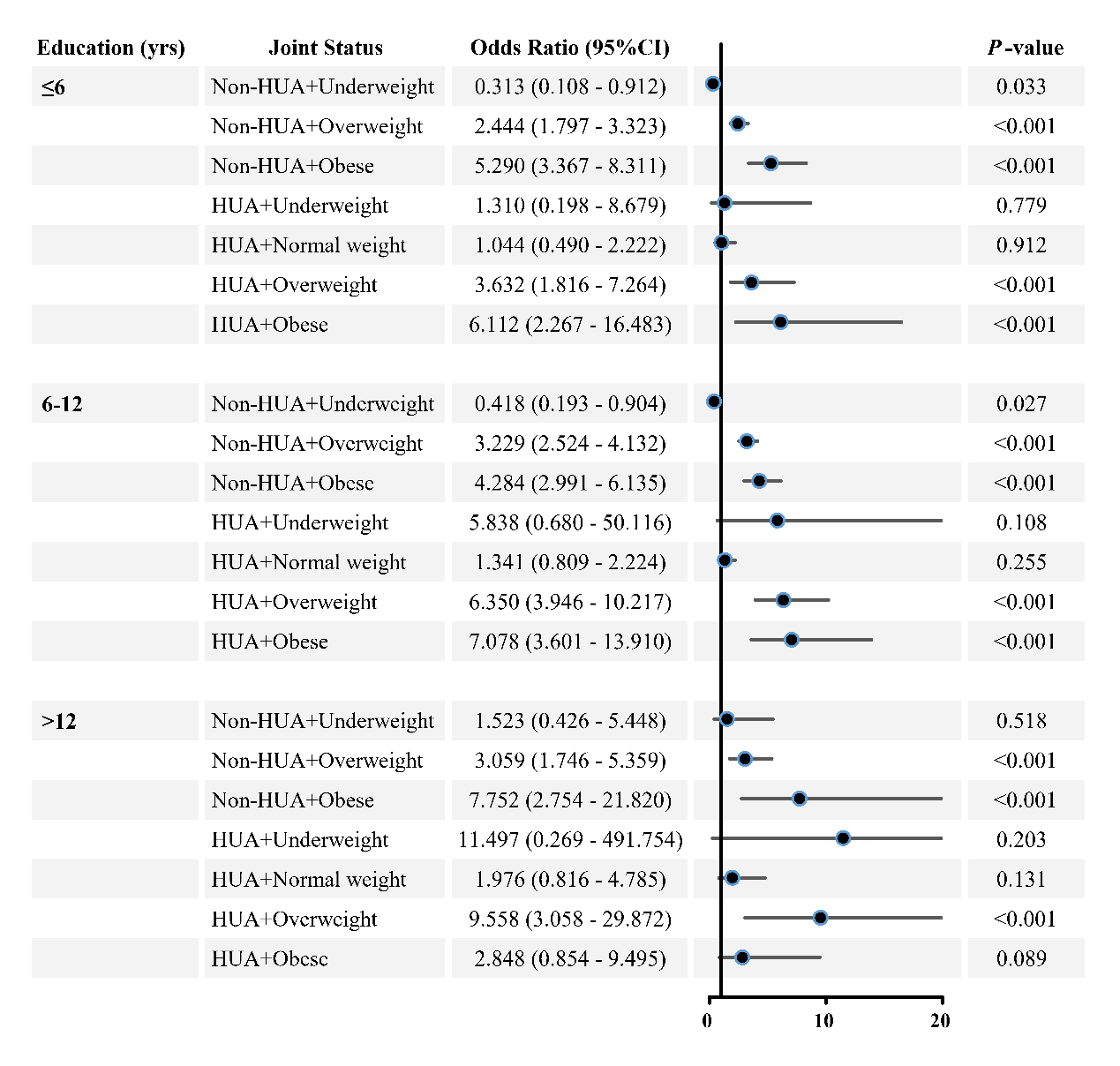
**

**D.2**

**
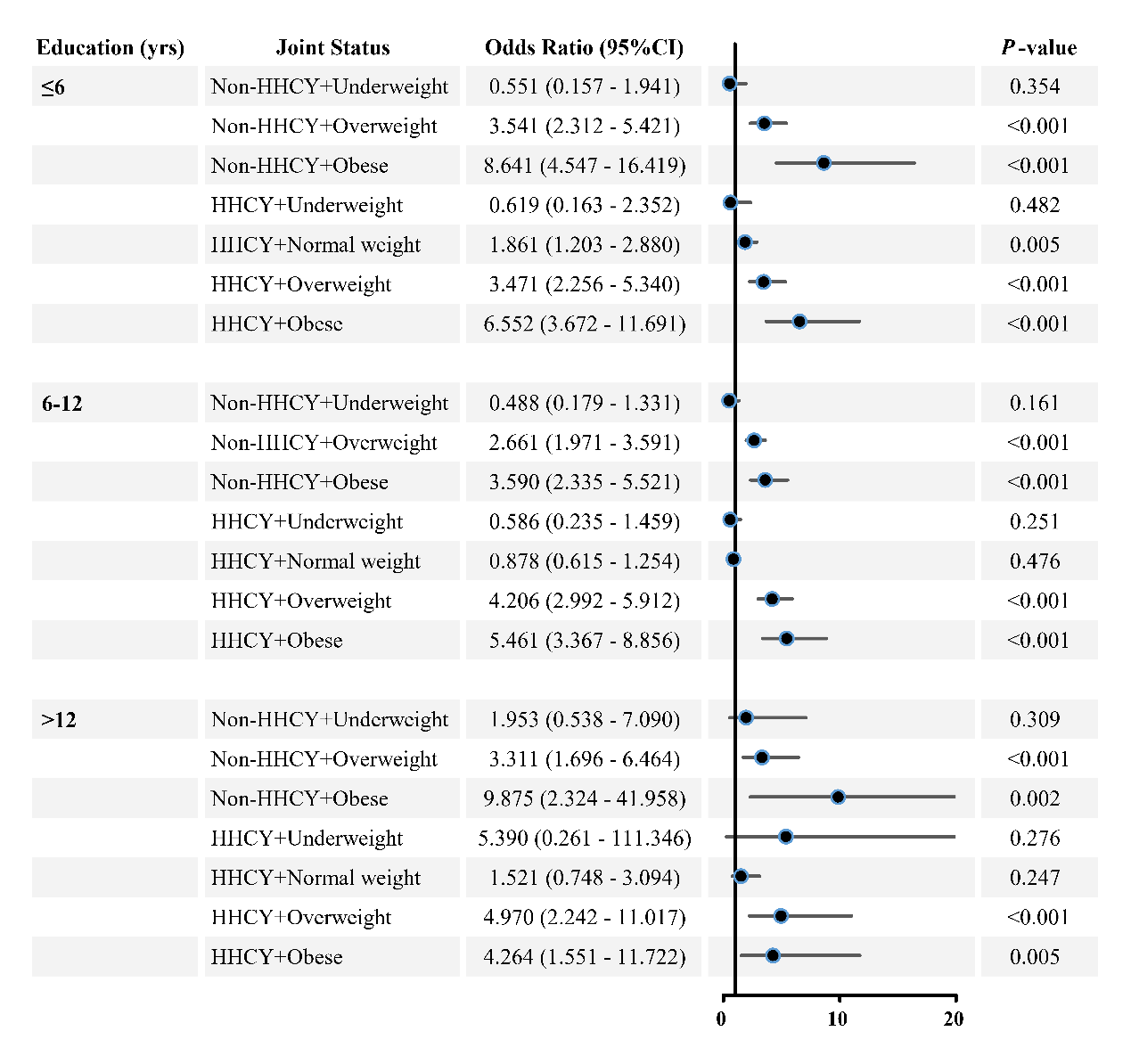
**

**D.3**

**
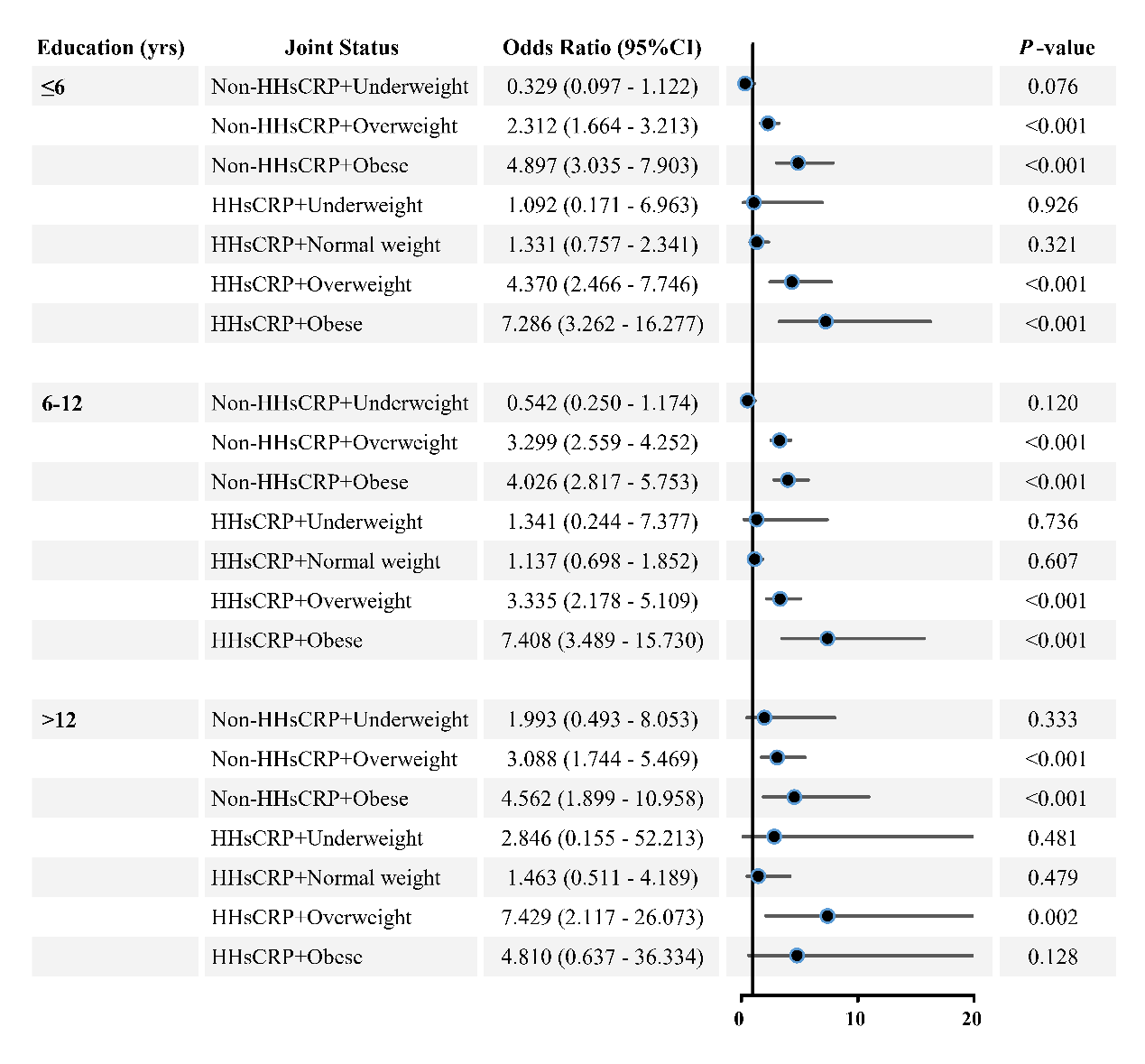
**

**E.1**

**
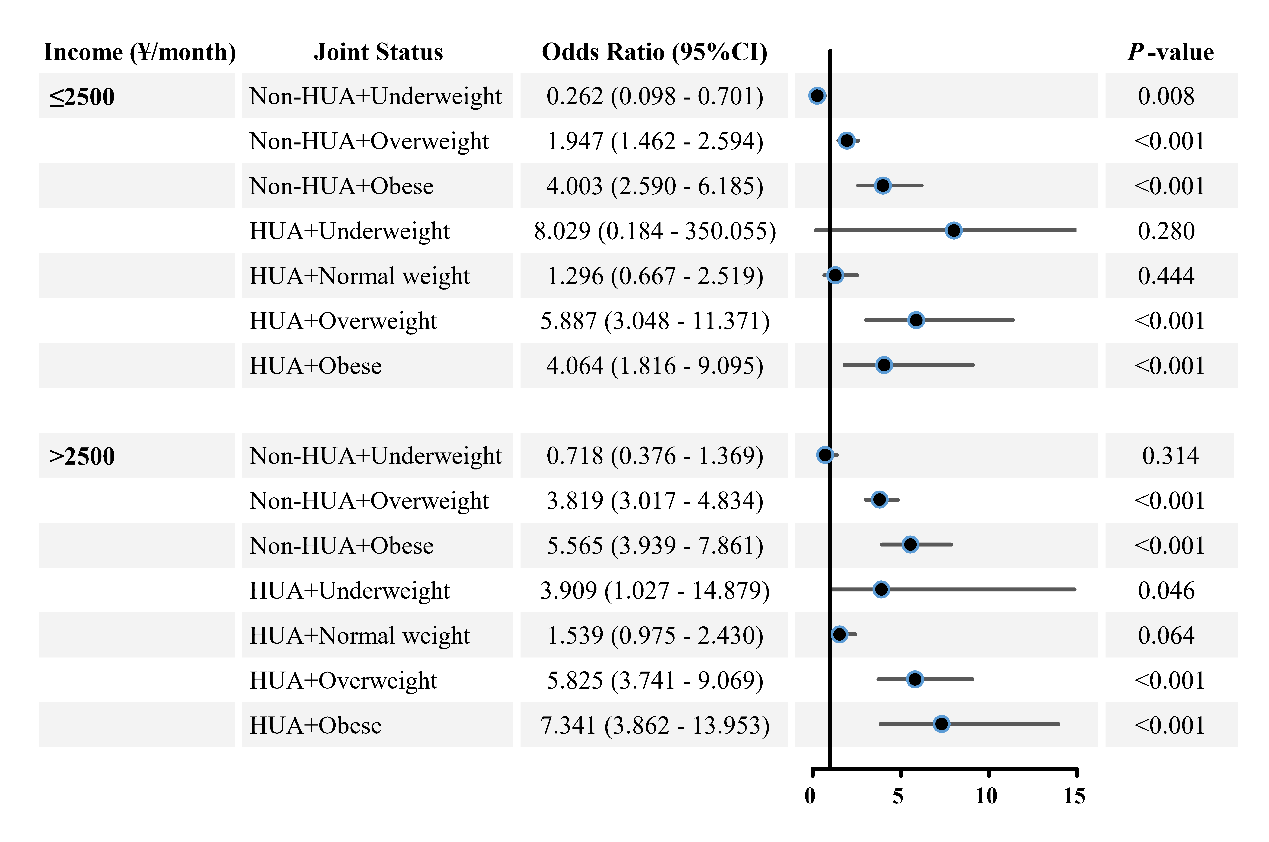
**

**E.2**

**
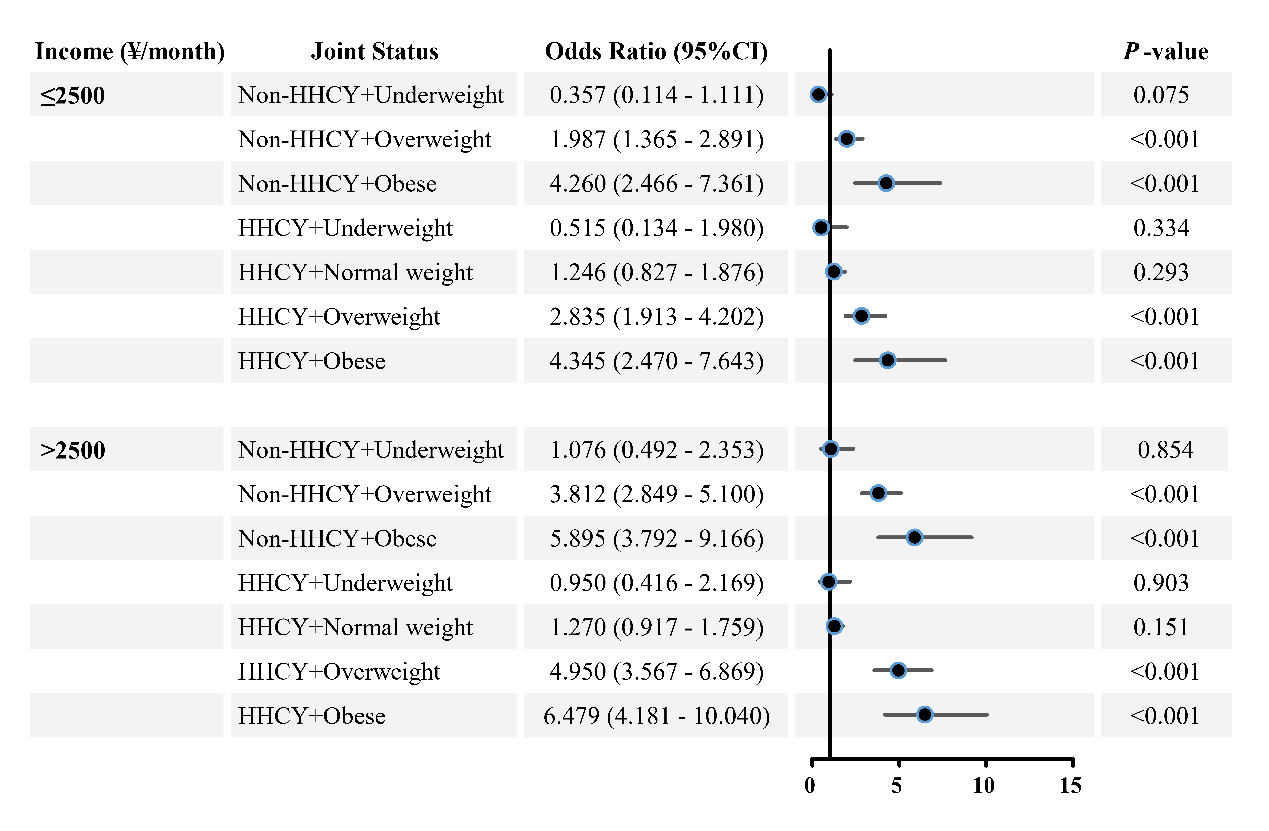
**

**E.3**

**
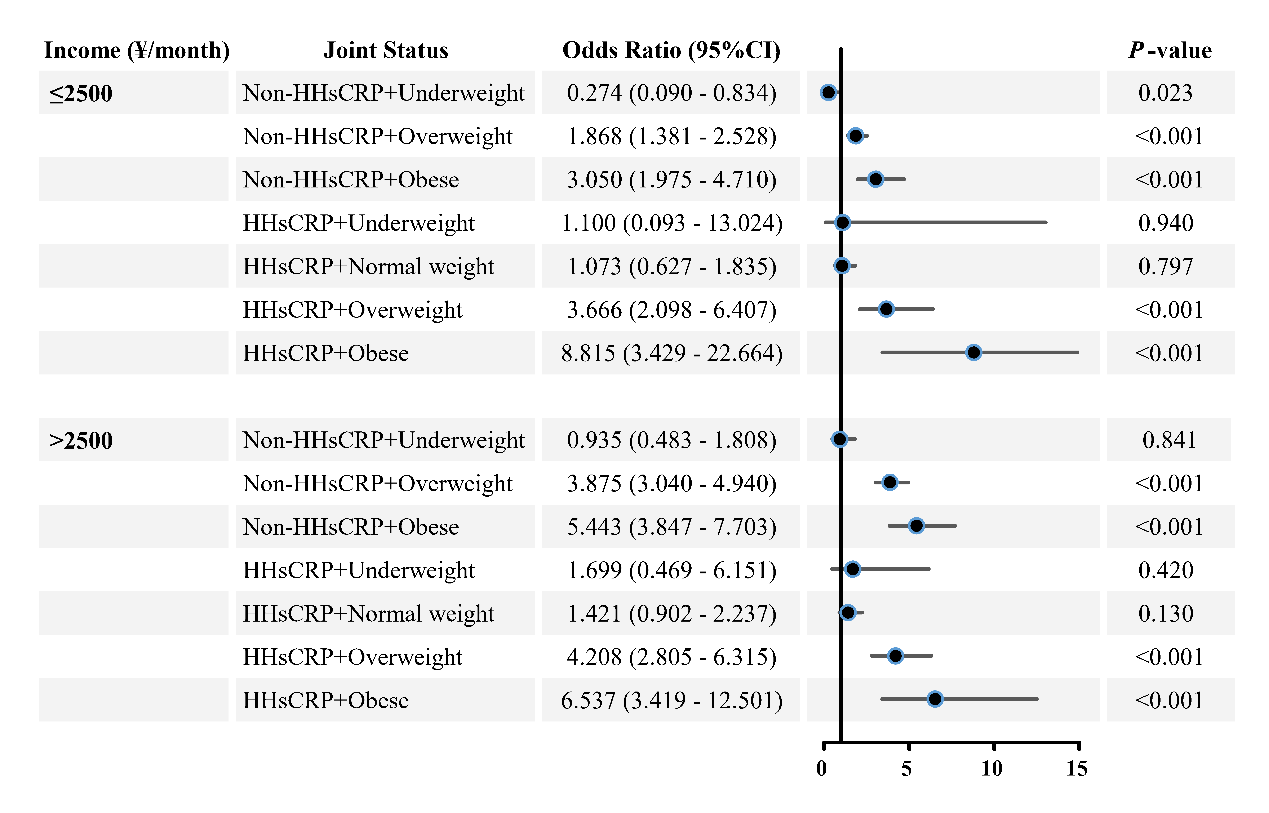
**
